# Out-of-Pocket Expenditure for Antenatal Care Amid Free Health Care Provision: Evidence From a Large Pregnancy Cohort in Rural Sri Lanka

**DOI:** 10.9745/GHSP-D-22-00410

**Published:** 2023-10-30

**Authors:** Sajan Praveena Gunarathna, Nuwan Darshana Wickramasinghe, Thilini Chanchala Agampodi, Indika Ruwan Prasanna, Suneth Buddhika Agampodi

**Affiliations:** a Department of Community Medicine, Faculty of Medicine and Allied Sciences, Rajarata University of Sri Lanka, Saliyapura, Sri Lanka.; b Department of Economics, Faculty of Social Sciences and Humanities, Rajarata University of Sri Lanka, Mihintale, Sri Lanka.; c Center for Public Health, Anuradhapura, Sri Lanka.

## Abstract

**Introduction:**

Global evidence suggests that high out-of-pocket (OOP) expenditure negatively affects health service utilization and creates an economic burden on households during pregnancy. This study aimed to estimate the magnitude and associated factors of OOP expenditure for antenatal care (ANC) in a rural Sri Lankan setting by following up with a large pregnancy cohort (The Rajarata Pregnancy Cohort [RaPCo]) in Anuradhapura District, Sri Lanka.

**Methods:**

Data were collected from July 2019 to May 2020. An interviewer-administered questionnaire was used to collect socioeconomic data and OOP expenditures in the first trimester. Self-administered questionnaires were used monthly to collect OOP expenditures in the second and third trimesters. In-depth financial information of 1,558 pregnant women was analyzed using descriptive statistics, nonparametric statistics, and a multiple linear regression model.

**Results:**

The majority of participants used both government and private health facilities for ANC. The mean (standard deviation [SD]) OOP expenditure per ANC visit was US$4.18 (US$4.19), and the mean (SD) OOP expenditure for total ANC was US$57.74 (US$80.96). Pregnant women who used only free government health services also spent 28% and 14% of OOP expenditure on medicines and laboratory investigations. Household income (*P*<.001), household expenditure (*P*<.1), used health care mode (*P*<.05), maternal morbidities (*P*<.05), and the number of previous pregnancies (*P*<.1) were the statistically significant independent predictors of OOP expenditure. OOP expenditure per visit for ANC equals half of the daily household expenditure.

**Conclusion:**

Despite having freely available government health facilities, most pregnant women tend to use both government and private health facilities and incur higher OOP expenditure. Free government health care users also incur a direct medical OOP expenditure for medicines and laboratory investigations. Monthly household income, expenditure, used health care mode, maternal morbidities, and the number of previous pregnancies are independent predictors of OOP expenditure.

## Abstract (precis)

The authors report that even with the free health care services provided by the government, out-of-pocket expenditure for antenatal care in Sri Lanka is high, and women in low-income groups have a higher expenditure compared to higher-income groups.

## Teaser

**Key Findings:**

- Global evidence suggests that high out-of-pocket (OOP) expenditure negatively affects health care utilization even with free health services in many low- and middle-income countries.
- Despite having access to free maternal health services provided by the government of Sri Lanka, most pregnant women tend to use both government and private health facilities and incur higher OOP expenditures.
- Because Sri Lanka provides free maternal health care services, the direct medical OOP expenditure for medicines and laboratory investigations should be at a minimum level. However, the direct medical OOP was approximately half of the OOP expenditure share for pregnant women who used only government health care services.

**Key Implications:**

- Use of free, government-provided maternal health care services should be encouraged to minimize the economic burden of maternal OOP expenditure, especially for low-income groups.
- Policies should be developed that ensure the adequate availability of medicines and essential services in government health care facilities.
- Future research should focus on the economic impact of high OOP expenditure on the households of pregnant women.

## INTRODUCTION

Payments made by families and individuals for medical services are known as out-of-pocket (OOP) expenditures. The World Bank defines OOP expenditure as^1^^,^^2^:

*Any direct payout by households, including gratuities and in-kind payments, to health care providers of pharmaceuticals, therapeutic appliances, and other goods and services whose primary intent is to contribute to the restoration or enhancement of the health status of individuals or population groups.*

In the global health agenda, OOP expenditure is considered a vital issue due to its negative consequences on individuals, families, and society. It leads individuals and families to poverty and indebtedness; reduces accessibility, affordability, and utilization of health services; and creates inequities in health.^3^ High OOP expenditure is a challenge to any country seeking to enhance the population's health in the most effective manner possible given the available resources and competing needs.^4^^–^^7^

The influence of OOP expenditure is a significant concern in maternal health services. OOP expenditure in maternal health care occurs as direct monetary payments for antenatal care (ANC), delivery, and postnatal care.^4^^,^^5^^,^^8^^,^^9^ The OOP expenditure for maternal care is divided into direct medical and nonmedical expenses.^6^^,^^10^^–^^12^

Global evidence indicates a relatively higher OOP expenditure for health care in low- and middle-income countries (LMICs) than in high-income countries.^13^ In most high-income countries, health care costs are largely covered by insurance plans,^14^ whereas LMICs rely heavily on OOP expenditure.^15^^,^^16^ The negative impact of OOP expenditure can be devastating when it leads to catastrophic expenditure, pushing families into economic vulnerability during pregnancy, especially in poor economic conditions in LMICs.^17^^,^^18^ A plethora of global literature reports the varying degrees of OOP expenditure for ANC, delivery, and postnatal care in LMICs.^19^^–^^25^ Evidence on the magnitude of OOP expenditure for ANC is scarce compared to the evidence on OOP expenditure for delivery and postpartum care.^26^ A recent systematic review and meta-analysis reported that the magnitudes of OOP expenditure for total ANC and per-visit ANC in LMICs are US$63.29 and US$12.93 in 2019 prices, respectively, and the total OOP expenditure for ANC ranged from US$2.41 to US$654.32 in LMICs.^25^ Compared with the other countries, the highest OOP expenditure for per-visit ANC was reported in Malawi (US$194.37) and the lowest was reported in Tanzania (US$2.65).^25^ Additionally, the amount of OOP expenditure for maternal morbidities during pregnancy in LMICs is reported highest in India (US$523.03) and lowest in Ethiopia (US$125.98). Evidence suggests that health service utilization is reduced due to high OOP expenditure in many African^16^^,^^27^^–^^30^ and South Asian^31^^,^^32^ countries. A high OOP expenditure for maternal care has negatively affected the free health policy and national incentive programs in many LMICs.^27^^,^^33^^,^^34^

Sri Lanka is a lower-middle-income country^35^ that provides health services free of charge at service delivery.^36^ However, approximately 40% of health expenditure is attributed to OOP expenditure in the Sri Lankan health care system.^37^ The country's maternal health care is well developed, and Sri Lanka is in the fourth phase of the obstetric transition.^38^^,^^39^ Pregnant women in Sri Lanka use the government health system more often than the private health system during pregnancy.^39^ A few studies have assessed the economic burden of ANC^40^^,^^41^ and postnatal morbidities,^42^ economic impact on pregnant women during COVID-19,^43^ and the impact of social determinants and economic factors on maternal deaths^44^ and maternal nutrition cost^45^^–^^48^ in rural Sri Lanka. Recent evidence suggests that, despite having access to free maternal health services, pregnant women have substantial OOP expenditure, even while using government health facilities in Sri Lanka.^40^

Despite having access to free maternal health services, pregnant women have substantial OOP expenditure, even while using government health facilities in Sri Lanka.

Despite a wealth of published global literature on different aspects of OOP expenditure related to maternal health care, evidence on this topic in Sri Lanka is scant. Further, detailed evidence is lacking on the magnitude of OOP expenditure pertaining to different events during the pregnancy period (e.g., pregnancy identification, clinic visits, health care seeking for maternal morbidities and hospitalization, etc.) and on the expenditure breakdown (e.g., direct medical and nonmedical OOP expenditure). A comprehensive analysis of OOP expenditure related to maternal health in a country with a free health care system would yield valuable insights on what aspects of health care should be addressed to reduce OOP expenditure for maternal health care, which ultimately promotes the accessibility, affordability, and utilization of health care services. Against this backdrop, this study aimed to determine the magnitude and associated factors of OOP expenditure for ANC in a selected rural Sri Lankan setting.

## METHODS

### Study Design

This study was a follow-up study of the pregnant women enrolled in the Rajarata Pregnancy Cohort (RaPCo), the largest pregnancy cohort as of 2022 in Sri Lanka.^49^ The RaPCo study was a prospective study conducted by the Rajarata University of Sri Lanka that aimed to determine (1) the etiologic associations of mental health during pregnancy with pregnancy outcomes, neonatal outcomes, and early childhood health and development outcomes and (2) the possible interactions of complex social issues, social capital, socioeconomic position, personality, and gender-based violence on health-related issues during pregnancy, pregnancy outcomes, neonatal outcomes, and early childhood outcomes.^49^ The study setting, period, population, and sample details are presented in relevant sections of this article and published elsewhere.^49^^,^^50^

The Sri Lankan health care sector provides essential maternal and child health services free of charge by the government (Table 1).^39^ However, due to overcrowding, time consumption, and inconvenience, some pregnant women seek private health care services for laboratory investigations, specialist care, and selected assessments.^40^ All the pregnant women enrolled in the RaPCo study received all routine assessments and a number of additional services free of charge (Table 1).^49^^,^^51^

**TABLE 1.** Laboratory Investigations and Assessments Provided Free for Rajarata Pregnancy Cohort Study Participants, Anuradhapura District, Sri Lanka^a^

	Weeks	
	0–12	25–28
Free services provided by government		
Examinations		
General examination and auscultation of heart	X	X
Obstetric examination	X	X
Blood pressure	X	X
Anthropometric assessment		
Height, weight, and BMI	X	N/A
Biochemical investigations		
Urinary protein and glucose	X	X
Screening for syphilis (VDRL) and HIV	X	N/A
Hemoglobin	X	X
Two-hour postprandial blood glucose	X	N/A
Oral glucose tolerance test	N/A	X
Paid services provided by private facilities		
Examinations		
Detailed cardiovascular system examination	X	X
2D echocardiography for selected women	N/A	X
Electrocardiogram for selected women	N/A	X
Anthropometric assessment		
Waist/hip ratio	X	N/A
Biochemical investigations		
Liver function tests	X	N/A
Ultrasound scan liver for selected subsample	X	N/A
Serum cholesterol	X	N/A
Oral glucose tolerance test	X	X
Full blood count	X	X
Blood picture	X	N/A
Serum ferritin, high performance liquid chromatography in anemic women	N/A	X
Serum cortisol	N/A	X
Other assessments and management		
Mental health assessment and management	X	X
Economic assessment	X	X

Abbreviations: 2D, two-dimensional; BMI, body mass index; N/A, not applicable; VDRL, venereal disease research laboratory.

a The investigations provided by the Rajarata Pregnancy Cohort are marked as “x.”

A larger prospective economic evaluation under the RaPCo study has been conducted to examine the economic burden of OOP expenditure, productivity loss of pregnant women, and the impact of the COVID-19 pandemic on the household economy of pregnant and postpartum women in rural Sri Lanka.^50^ The complete economic evaluation was a follow-up study conducted throughout the pregnancy period from the time of pregnancy identification to delivery. The present study is a subcomponent (economic burden of OOP expenditure) of the economic evaluation of the RaPCo study. The cohort profile, study protocols of the RaPCo, and the economic study present more details on the methods and the findings, respectively.^49^^–^^51^

### Study Setting

The study was conducted in the Anuradhapura district, the largest district in Sri Lanka (7,179 km^2^). The district population is 902,930, with a birth rate of 17.8/1,000 population. The majority (92.7%) live in rural areas, and the primary income source is agriculture-based, reporting a median monthly household income of US$285.91.^52^ The total health expenditure comprises 58% of the government and private health care sectors in the Anuradhapura district.^53^

**FIGURE 1:**
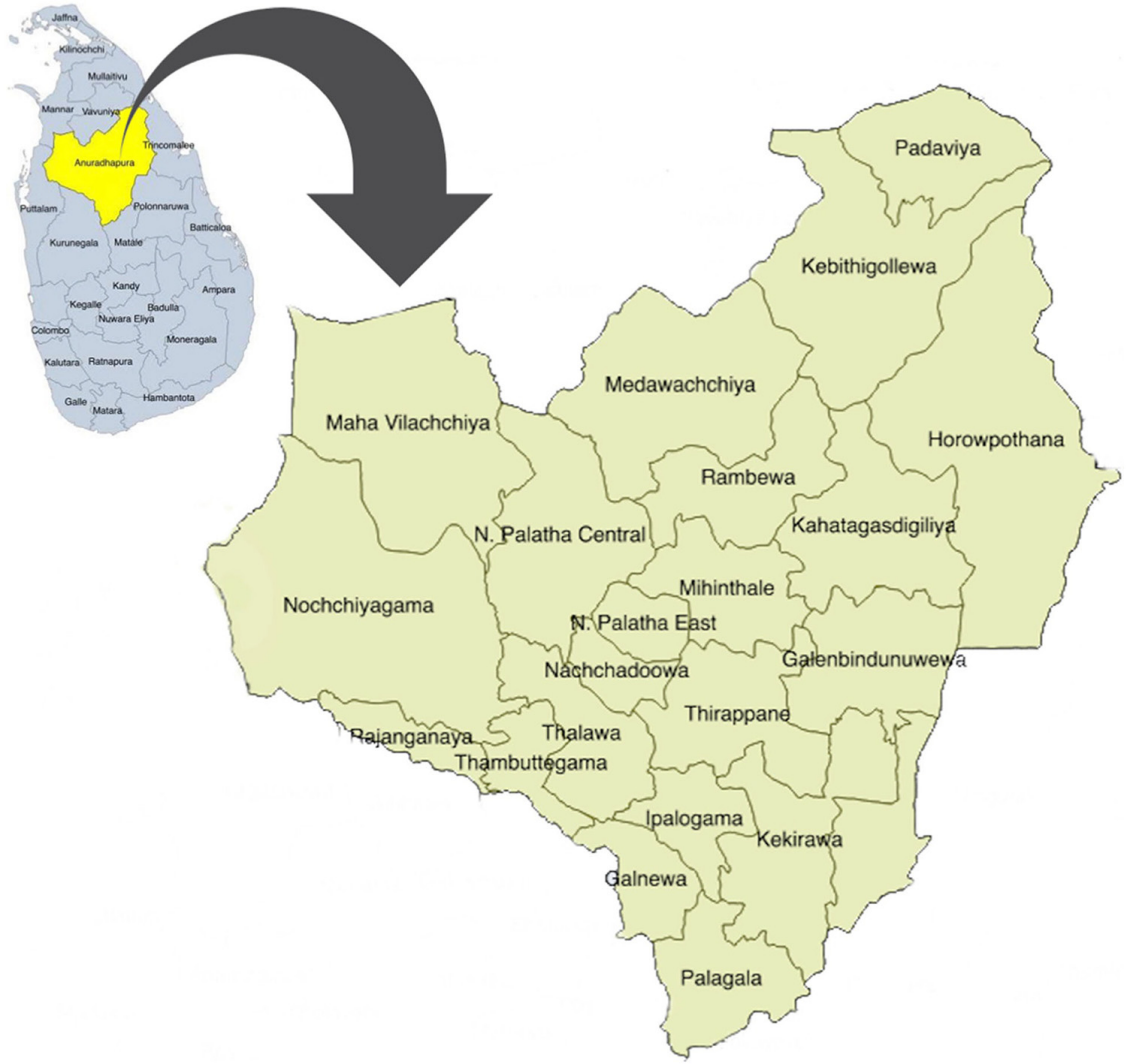
Map of 22 Participant Recruitment Areas of the Rajarata Pregnancy Cohort Study, Anuradhapura District, Sri Lanka^a^ ^a^ Reproduced with permission from Agampodi et al.^49^

In Sri Lanka, the government provides field ANC free of charge through the medical officer of health (MOH) and public health midwife (PHM). In line with the national ANC program, the ANC services in the Anuradhapura District are provided through 22 MOH areas and 275 PHM areas (Figure 1). Approximately 17,000 pregnant women register annually at the PHM, and the number of live births is around 15,000.^49^ Almost 96.0% of pregnant women visit at least 1 clinic provided by the government's free health care system. PHM provides care through domiciliary visits and clinical care at the antenatal clinics conducted in the MOH office. The district comprises 56 hospitals, including a teaching hospital and 3 base hospitals for curative health services. Most deliveries (99.5%) are placed in government health facilities.^49^

### Study Population and Sample

The study population included pregnant women registered in the maternal health care program in Anuradhapura district, Sri Lanka, where the ANC coverage is 82.3%.^49^ All eligible pregnant women included in the RaPCo study from July 1, 2019 to September 30, 2019 were invited to participate in this study. The original cohort included more than 90.0% of the study population during the study period.^49^^,^^51^

The eligibility criteria were pregnant women who were registered at the PHM and visiting antenatal field clinics in the Anuradhapura district, whose permanent residence was in the Anuradhapura district and had not planned to leave the district until their delivery, and whose period of amenorrhea/gestational age was less than 12 weeks by the time of recruitment.

Pregnant women who planned to leave the study setting for delivery and with uncertain due dates were excluded from the study. The recruitment of pregnant women for the study was done with the help of PHMs in each MOH area by organizing a special clinic under the permission of the public health authorities in the district. Participants gave their informed written consent before the study. Ethical approval and administrative clearance were given before the recruitment.

### Data Collection

The baseline data were collected at the recruitment of study participants using an interviewer-administered questionnaire, and 22 trained data collectors were employed, with 1 for each MOH area. The questionnaire included sociodemographic, economic, and health-related information and in-depth financial details for the first trimester of pregnancy. A self-administered questionnaire was used to collect monthly in-depth financial details related to ANC during the second and third trimesters. All the study tools were pretested among a sample of pregnant women in the Anuradhapura district who did not belong to the RaPCo study. The questionnaires were edited according to the suggestions of the pretesting, and all the study tools have been published previously along with the study protocol.^50^

We provided verbal and written instructions on how and when to complete the questionnaires. We asked pregnant women to keep the expenses in a diary to enhance the accuracy before completing the questionnaire. Pregnant women who were unaware of the payments made for ANC were asked to fill out the questionnaire with the support of the spouse/family member and informed to contact the research team where necessary. Phone reminders were given monthly to minimize attrition.

### Definition of Measures: Magnitude of OOP Expenditure

The World Bank definition of OOP expenditure was used to quantify the magnitude of OOP expenditure in the study.^2^ When adjusting the World Bank definition to maternal health care, OOP expenditure includes all direct monetary expenditures incurred by a pregnant woman or her family on maternal health care. The detailed breakdown of OOP expenditure included direct medical and nonmedical expenses.^54^

- Direct medical OOP expenditure: cost for consultation, laboratory investigation, medicines, hospital charges, and other direct medical payments for maternal health care.
- Direct nonmedical OOP expenditure: cost of traveling, food, and refreshments during health-seeking activity, both for the pregnant woman and for accompanying person/s.

We considered all these cost categories at pregnancy identification, first and subsequent clinic visits, health-seeking activity for maternal morbidities/ill-health conditions, and hospitalization for maternal morbidities or any related health condition.

The health care modes used by pregnant women were categorized as government health care services, private health care services, and both government and private health care services. The category of “both government and private health care services” included pregnant women who made at least 1 visit to each type of facility. We also assessed the cost incurred to treat maternal ill-health conditions. The reported maternal ill-health conditions were (1) shortness of breath, chest tiredness, and chest pain; (2) diagnosed hypertension complicating pregnancy; (3) shortness of breath, cough, and cold; (4) dysuria, oliguria, and lower abdominal pain; (5) nausea and vomiting; (6) swelling of the legs/varicose vein; (7) vaginal bleeding; (8) headache/faintness; (9) fever; (10) vaginal discharge and itching; (11) severe abdominal pain; (12) diagnosed anemia; (13) diagnosed hyperglycemia in pregnancy; (14) diseases related to the thyroid gland; (15) backache; (16) constipation; and (17) other illnesses. The income categories used for the analysis included low-, middle-, and high-income groups. These groups were created using the quintile approach, and we amalgamated lower-middle and upper-middle-income categories as the middle-income group.

### Data Analysis

Data entering, cleaning, and management were done using Microsoft Excel, and data analysis was carried out using SPSS V27. We double-checked the incompatible entries with the corresponding questionnaire and corrected them before the analysis. The magnitude of OOP expenditure was computed as the cost per-visit basis. We estimated the total OOP expenditure using per-visit OOP expenditure:

Total OOP expenditure_i_ = per-visit OOP expenditure_i_ × number of visits_i_

where i is the pregnant woman

Families facing catastrophic expenditures were estimated using OOP expenditure and household expenditure per month according to the threshold level of 10% of household expenditure.

All the monetary values were converted to the average exchange rate (US$ to Sri Lankan Rupees [LKR]) of the data collection period from July 2019 to May 2020 (i.e., US$1=LKR182.30).^55^ The average exchange rate for this period was based on the fact that there were no outliers, and the standard deviation was 4.33 only. However, sensitivity analysis was done by presenting the magnitude of OOP expenditure with the reported highest (US$1=LKR191.67) and lowest (US$1=LKR175.99) exchange rates within the period.^55^

Before analysis, we checked whether the present study's sample matched the RaPCo sample using important and relevant baseline variables: religion, ethnicity, education level, and the status of reproductive health education. Sample characteristics (including sociodemographic, economic, and health-related information), magnitude of OOP expenditure, and sources of financing for OOP expenditure are presented using univariate descriptive statistical measures. Before the bivariate analysis, we tested whether the data were normally distributed using the Shapiro-Wilk test, and the results revealed that the data was not normally distributed (*P*<.05). Thus, nonparametric statistical tests were used for data analysis, including the Friedman, Wilcoxon Signed Ranks, Kruskal-Wallis, Mann-Whitney U, and Spearman Rank Correlation.

Further, we used the multiple linear regression model (MLRM) to assess the independent predictors of OOP expenditure, identified in the literature and as statistically significant in the bivariate analysis. In addition, residual analysis (Durbin-Watson D statistics and variance inflation factor) was performed to detect autocorrelation and multicollinearity. We used MLRM because the standardized and unstandardized residuals are normally distributed according to the Shapiro-Wilk Statistics (statistic=0.984 [degree of freedom=1,226]; *P*=.247). Also, the study has a higher sample size. According to Knofczynski and Mundfrom, the present study's MLRM is adequate to get an excellent prediction according to the sample size recommendations at selected levels of squared population multiple correlation coefficients (R-squared) for performing MLRM.^56^

**FIGURE 2:**
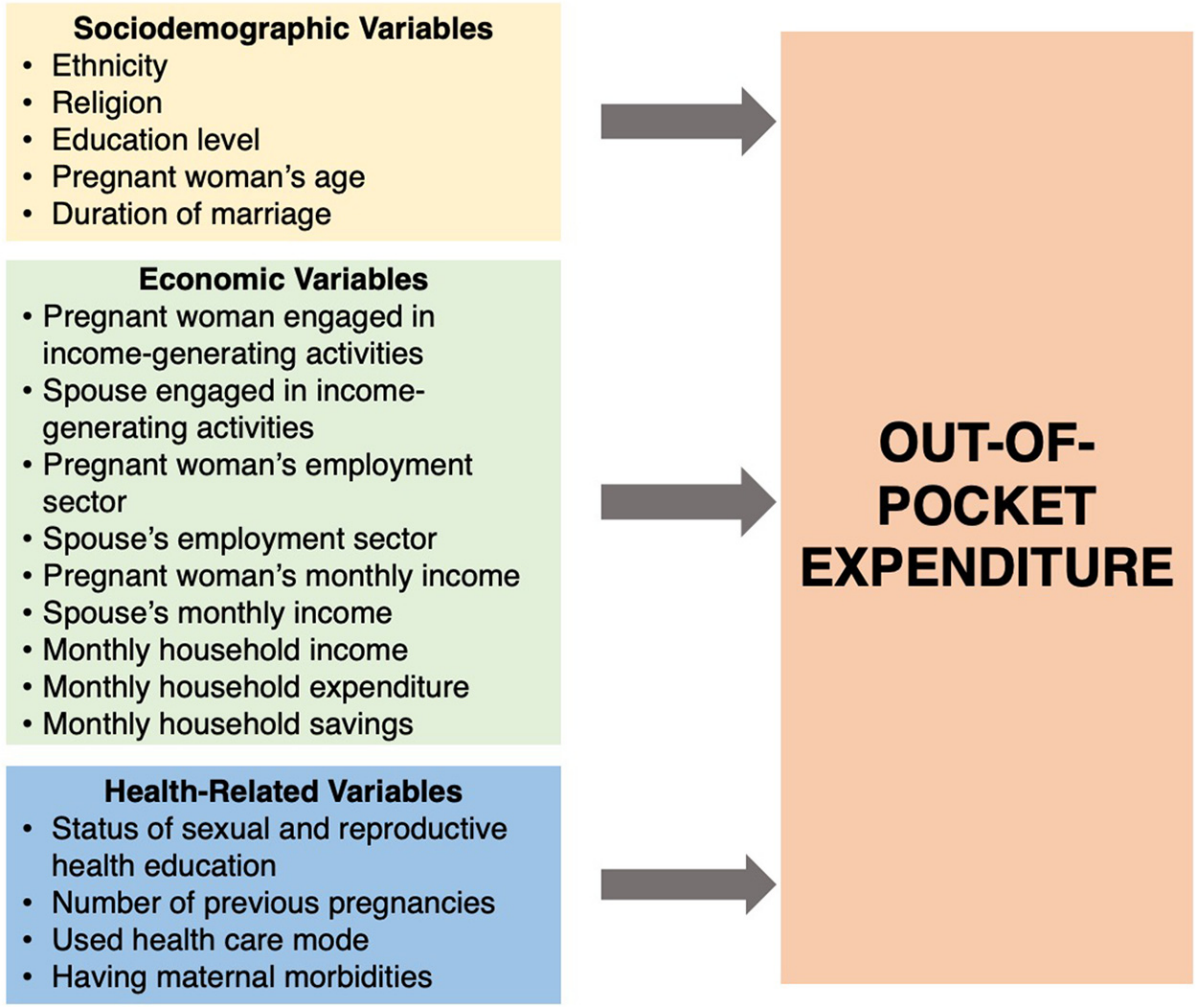
Conceptual Framework of Independent Predictors of Changes in Out-of-Pocket Expenditure for Antenatal Care in Health Care Facilities, Anuradhapura District, Sri Lanka

The hypothesis we intended to test was “whether changes in selected independent predictor/s are associated with changes in OOP expenditure.” The selected independent predictors included 18 variables: 5 sociodemographic variables, 9 economic variables, and 4 health-related variables. All the selected variables are presented in the conceptual framework (Figure 2).

### Ethical Approval

This study is a part of the RaPCo study, and the ethical approval for the RaPCo study was obtained from the ethics review committee, Faculty of Medicine and Allied Sciences, Rajarata University of Sri Lanka (ERC 2019/07).

## RESULTS

We invited all pregnant women of the RaPCo study (N=3,074) to participate in the economic study, and 1,558 women participated in the present study (response rate=50.7%). Because the RaPCo study sample was closer to the population during the study period, we compared whether the economic study sample's selected sociodemographic variables differed from the pregnant women who did not participate. We identified that the household income (*P*=.743), household expenditure (*P*=.512), religion (*P*=.363), ethnicity (*P*=.515), education level (*P*=.361), and status of sexual and reproductive health education (*P*=.344) were not significantly different between the present study sample participants and others in the RaPCo study.

### Sample Characteristics

Of the 1,558 pregnant women who were initially enrolled in the study, in-depth financial details related to ANC were completed by 1,425 (91.5%), 1,286 (82.5%), and 424 (27.2%) pregnant women in the first, second, and third trimesters, respectively.

Table 2 presents the sample characteristics according to socioeconomic, demographic, and health-related factors. The mean (standard deviation [SD]) age of the pregnant women was 28.3 (5.6) years. Most pregnant women were Sinhalese (1,351 [89.6%]), Buddhist (1,337 [88.6%]), and had studied up to the senior secondary level (1,198 [79.9%]). The mean (SD) marriage duration was 6.0 (4.7) years.

**TABLE 2.** Sociodemographic, Economic, and Health Characteristics of Rajarata Pregnancy Cohort Study Sample Participants, Anuradhapura District, Sri Lanka

Characteristic	No. (%)
Ethnicity (n=1,508, 96.8%)^a^	
Sinhalese	1,351 (89.6)
Tamil	9 (0.6)
Moor	143 (9.5)
Malays	1 (0.1)
Other	4 (0.3)
Religion (n=1,509, 96.9%)^a^	
Buddhist	1,337 (88.6)
Catholic/Christian	20 (1.3)
Hindu	5 (0.3)
Islam	147 (9.7)
Educational status (n=1,500, 96.3%)^a^	
Primary	15 (1.0)
Junior secondary	63 (4.2)
Senior secondary	1,198 (79.9)
Higher education	224 (14.9)
Marital status (n=1,448, 92.9%)^a^	
Mean (SD), years married	6.0 (4.7)
Median (IQR)	5.0 (1.7–9.0)
Age of pregnant woman, years (n=1,497, 96.1%)^a^	
Mean (SD)	28.3 (5.6)
Median (IQR)	28 (25–32)
Engaged in income-generating activity (n=1,393, 89.4%)^a^	
No	1,098 (78.8)
Yes	295 (21.2)
Sector of employment, (n=295, 18.9%)^a^	
Government	112 (38.0)
Private	82 (27.8)
Other^b^	101 (34.2)
Monthly income of pregnant woman, US$ (n=295, 18.9%)^a^	
Mean (SD)	149.80 (122.10)
Median (IQR)	137.14 (82.28–196.93)
Engaged in income-generating activity (n=1,393, 89.4%)^a^	
No	86 (6.2)
Yes	1307 (93.8)
Sector of employment (n=1,299, 83.4%)^a^	
Government	421 (32.4)
Private	379 (29.2)
Other^b^	499 (38.4)
Monthly income of the pregnant woman's husband, US$ (n=1,307, 93.8%)^a^	
Mean (SD)	224.27 (157.96)
Median (IQR)	197.48 (164.56–246.85)
Monthly household income, US$ (n=1,393, 89.4%)^a^	
Mean (SD)	277.29 (216.04)
Median (IQR)	219.42 (164.56–318.16)
Income categories (n=1,356, 87.0%)^a^	
Low (<US$164.56)	357 (26.7)
Middle (US$164.57–US$318.16)	651 (48.6)
High (>US$318.17)	330 (24.7)
Monthly household savings, US$ (n=1,345, 86.3%)^a^	
Mean (SD)	50.85 (83.77)
Median (IQR)	27.43 (10.97–54.85)
Monthly household expenditure, US$ (n=1,296, 83.2%)^a^	
Mean (SD)	190.19 (103.11)
Median (IQR)	171.14 (123.15–231.08)
Status of sexual and reproductive health education (n=1,472, 94.5%)^a^	
Yes	885 (60.1)
No	617 (41.9)
Attendance of pre-pregnancy clinics (n=1,486, 95.4%)^a^	
Yes	1,099 (74.0)
No	387 (26.0)
Previous pregnancies, median (IQR) (n=1,528, 98.1%)^a^	2 (1–3)
Gestational age at recruitment, weeks (n=1,558, 100%)^a^	
Mean (SD)	9.1 (3.1)
Median (IQR)	8 (7–10)
Number of health visits, median (IQR) (n=1,473, 94.5%)^a^	
First trimester	3 (2–3)
Second trimester	3 (3–5)
Third trimester	6 (3–6)
During pregnancy	11 (9–14)
Used health care mode (n=1,529, 98.1%)^a^	
Government	409 (26.7)
Private	81 (5.3)
Both government and private	1,039 (68.0)
Having maternal morbidities/ill-health conditions (self-reported) (n=1,557, 99.9%)^a^	
No morbidities	107 (6.9)
Having only 1 morbidity	292 (18.8)
Having >1 morbidity	1,158 (74.4)

Abbreviations: IQR, interquartile range; SD, standard deviation.

a Number and percentage of pregnant women in terms of the total sample (1,558 pregnant women).

b Under the employment sector, the “other” group includes those who do not receive a monthly wage, but the income comes from daily earnings in labor work, agriculture sector, and other non-fixed earning groups.

According to the health-related information, the mean (SD) gestational age at the recruitment was 9.1 (3.1) weeks. The median (interquartile range [IQR]) for previous pregnancies was 2 (1–3), and the median (IQR) number of health visits during the pregnancy was 11 (9–14). Most pregnant women had used both government and private health care facilities (1,039 [68.0%]), while 409 (26.7%) pregnant women used only government health facilities and 81 (5.3%) used only private health facilities. Most pregnant women (1,158 [74.4%]) had more than 1 maternal morbidity/ill-health condition during pregnancy.

When considering the household economic background, only 295 (21.2%) pregnant women were involved in regular income-generating activities; of them, the majority (112 [38.0%]) were employed in the government sector. The mean (SD) and median (IQR) monthly household income were US$277.29 (US$216.04) and US$219.42 (US$164.56–US$318.16), respectively. Among the sample, 357 (26.7%) were in the low-income (<US$164.56) group, while 651 (48.6%) and 330 (24.7%) belonged to the middle-income group (US$164.57–US$318.16) and high-income group (>US$318.17), respectively. The mean (SD) and median (IQR) monthly household expenditures were US$190.19 (US$103.11) and US$171.14 (US$123.15–US$231.08), respectively.

### Magnitude of Per-Visit OOP Expenditure During Pregnancy and in Different Trimesters

The mean (SD) and median (IQR) per-visit OOP expenditure during pregnancy were US$4.18 (US$4.19) and US$3.11 (US$1.35–US$5.63), respectively (using the average exchange rate). The 75^th^, 50^th^, and 25^th^ percentiles of per-visit OOP expenditure were US$5.63, US$3.11, and US$1.35, respectively. The mean (SD) and the median (IQR) per-visit OOP expenditure during pregnancy, with the highest exchange rate reported, were US$3.98 (US$3.99) and US$2.95 (US$1.28–US$5.36), respectively. The mean (SD) and the median (IQR) per-visit OOP expenditure during pregnancy, with the lowest exchange rate reported, were US$4.33 (US$4.34) and US$3.22 (US$1.39–US$5.83), respectively.

The mean (SD) per-visit OOP expenditure was US$6.05 (US$7.82), US$6.05 (US$7.32), and US$7.37 (US$8.96) during the first, second, and third trimesters, respectively. The corresponding median values were US$3.96 (US$0.91–US$8.23), US$3.54 (US$0.91–US$8.78), and US$4.98 (US$0.91–US$10.50) (Table 3). The breakdown of the OOP expenditure according to different expenditure events is given in Supplement Table S1.

**TABLE 3.** Magnitude of Per-Visit Out-of-Pocket Expenditure During Pregnancy Among Rajarata Pregnancy Cohort Study Sample Participants, Anuradhapura District, Sri Lanka

	First Trimester			Second Trimester			Third Trimester			Total Pregnancy		
	No. (%) (N=1,558)	Mean (SD), US$	Median (IQR), US$	No. (%) (N=1,558)	Mean (SD), US$	Median (IQR), US$	No. (%) (N=1,558)	Mean (SD), US$	Median (IQR), US$	No. (%) (N=1,558)	Mean (SD), US$	Median (IQR), US$
Expenditure breakdown, by different expenditure means												
Direct medical out-of-pocket expenditure												
Medicine/micronutrient supplements	517 (33.2)	2.20 (3.31)	0.79 (0.24–2.74)	731 (46.9)	2.08 (2.96)	0.98 (0.40–2.56)	213 (13.8)	2.49 (3.57)	1.28 (0.55–2.93)	1,036 (66.5)	1.03 (1.49)	0.54 (0.16–1.28)
Consultation	381 (24.5)	6.84 (8.19)	6.86 (2.68–8.23)	638 (40.9)	5.60 (3.83)	5.12 (2.74–7.31)	207 (13.3)	7.30 (3.73)	7.13 (5.49–8.23)	905 (58.1)	2.83 (2.51)	2.38 (1.35–3.52)
Laboratory investigation	635 (40.8)	4.06 (3.50)	3.66 (2.21–5.30)	122 (7.8)	1.68 (2.08)	0.82 (0.46–1.92)	26 (1.7)	2.44 (1.82)	2.08 (1.04–3.84)	722 (46.3)	1.32 (1.18)	1.22 (0.61–1.77)
Hospital charges	11 (0.7)	2.45 (2.45)	1.83 (1.13–2.23)	10 (0.6)	4.53 (3.87)	2.74 (1.83–6.86)	0	-	-	21 (1.3)	1.72 (1.65)	1.01 (0.82–1.92)
Other direct medical costs^a^	897 (57.6)	0.22 (0.34)	0.14 (0.08–0.22)	104 (6.7)	2.43 (3.11)	1.51 (0.91–2.7)	25 (1.6)	6.35 (5.30)	5.49 (2.74–10.97)	946 (60.1)	0.22 (0.62)	0.05 (0.03–0.11)
Direct nonmedical out-of-pocket expenditure												
Travel	994 (63.8)	0.85 (1.54)	0.41 (0.19–0.82)	991 (63.6)	0.96 (1.28)	0.52 (0.24–1.10)	329 (21.1)	1.12 (1.84)	0.55 (0.27–1.01)	1,397 (89.7)	0.52 (0.69)	0.29 (0.12–0.62)
Food and refreshments	788 (50.6)	0.85 (1.48)	0.55 (0.27–0.91)	768 (49.3)	0.96 (1.16)	0.55 (0.32–1.19)	259 (16.6)	1.15 (1.46)	0.69 (0.41–1.37)	1,207 (77.5)	0.47 (0.60)	0.30 (0.15–0.60)
Accompanying person(s)	418 (26.8)	0.66 (0.89)	0.37 (0.18–0.80)	369 (23.7)	0.94 (1.30)	0.55 (0.27–1.10)	104 (6.8)	1.21 (1.49)	0.69 (0.43–1.37)	713 (45.8)	0.35 (0.51)	0.18 (0.09–0.41)
Other direct nonmedical costs^a^	265 (17.0)	1.07 (1.97)	0.27 (0.14–1.08)	156 (10.0)	0.96 (1.52)	0.37 (0.16–0.91)	54 (3.5)	1.33 (1.68)	0.62 (0.18–1.83)	424 (27.2)	0.40 (0.65)	0.13 (0.05–0.46)
Expenditure breakdown, by health care mode												
Government health care	572 (36.7)	1.59 (2.24)	0.77 (0.27–1.75)	453 (29.1)	2.52 (4.14)	1.23 (0.55–2.77)	221 (14.2)	2.53 (5.15)	0.96 (0.41–2.47)	395 (25.4)	1.07 (1.32)	0.63 (0.26–1.46)
Private health care	124 (8.0)	17.21 (11.01)	15.23 (11.46–20.18)	178 (11.4)	13.16 (8.23)	10.97 (8.78–15.13)	125 (8.0)	11.47 (6.40)	9.97 (7.82–13.27)	81 (5.2)	6.32 (4.38)	5.06 (3.61–8.11)
Both government and private health facilities^b^	672 (43.1)	8.06 (7.51)	6.56 (4.09–9.44)	462 (29.7)	8.96 (7.37)	6.74 (4.21–11.39)	78 (5.0)	14.51 (12.37)	10.79 (6.63–17.46)	1,039 (66.7)	5.20 (4.29)	4.05 (2.42–6.50)
Final OOP expenditure value	1,425 (91.5)	6.05 (7.82)	3.96 (0.91–8.23)	1,286 (82.5)	6.05 (7.32)	3.54 (0.91–8.78)	424 (27.2)	7.37 (8.96)	4.98 (0.91–10.50)	1,558 (100)	4.18 (4.19)	3.11 (1.35–5.63)

Abbreviations: IQR, interquartile range; OOP, out-of-pocket; SD, standard deviation; US$, United States dollar.

a Includes any medical-related items, human chorionic gonadotropin strips for pregnancy identification, and any other items purchased outside during hospitalization.

b Cost incurred for pregnant women who used both government and private health facilities.

The OOP expenditure per visit was 40.0% of per day household income (mean=US$9.24) and 52.1% per day household expenditure (mean=US$6.34). The OOP expenditure per visit was 47.7%, 40.3%, and 50.0% of per day household income in the first, second, and third trimesters, respectively. The shares of per-visit OOP expenditure were 63.6%, 58.8%, and 68.7% of per day monthly household expenditure in 3 trimesters, respectively. There was no statistically significant difference in per-visit OOP expenditure across 3 trimesters (χ^2^ [2]=4.306, *P*=.116). Further, there was no statistically significant difference in per-visit OOP expenditure share over per day income across 3 trimesters (χ^2^ [2]=3.349, *P*=.187).

**FIGURE 3:**
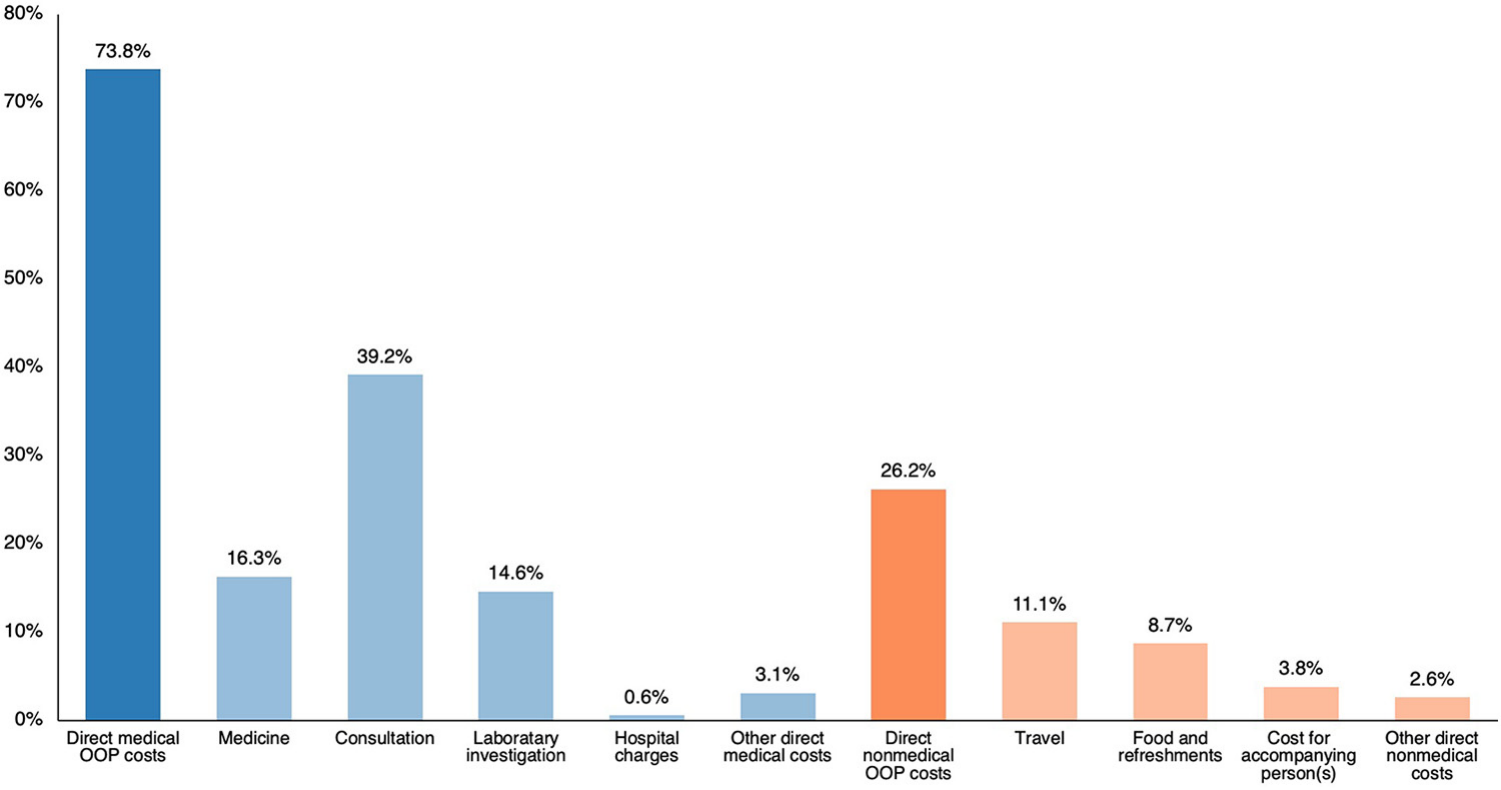
Distribution of Out-of-Pocket Expenditure for Antenatal Care in Health Care Facilities, Anuradhapura District, Sri Lanka Abbreviation: OOP, out-of-pocket.

According to the expenditure breakdown, per visit, direct medical OOP expenditure (73.8% of total OOP expenditure) was higher than the direct nonmedical OOP expenditure (26.2% of total OOP expenditure) (Figure 3), and it was statistically significant (Z=−25.240, *P*=.001). Further, per visit, direct medical OOP expenditure was higher than the direct nonmedical cost in the first trimester (Z=−19.056, *P*=.001), second trimester (Z=−18.952, *P*=.001), and third trimester (Z=−10.746, *P*=.001) respectively.

**FIGURE 4:**
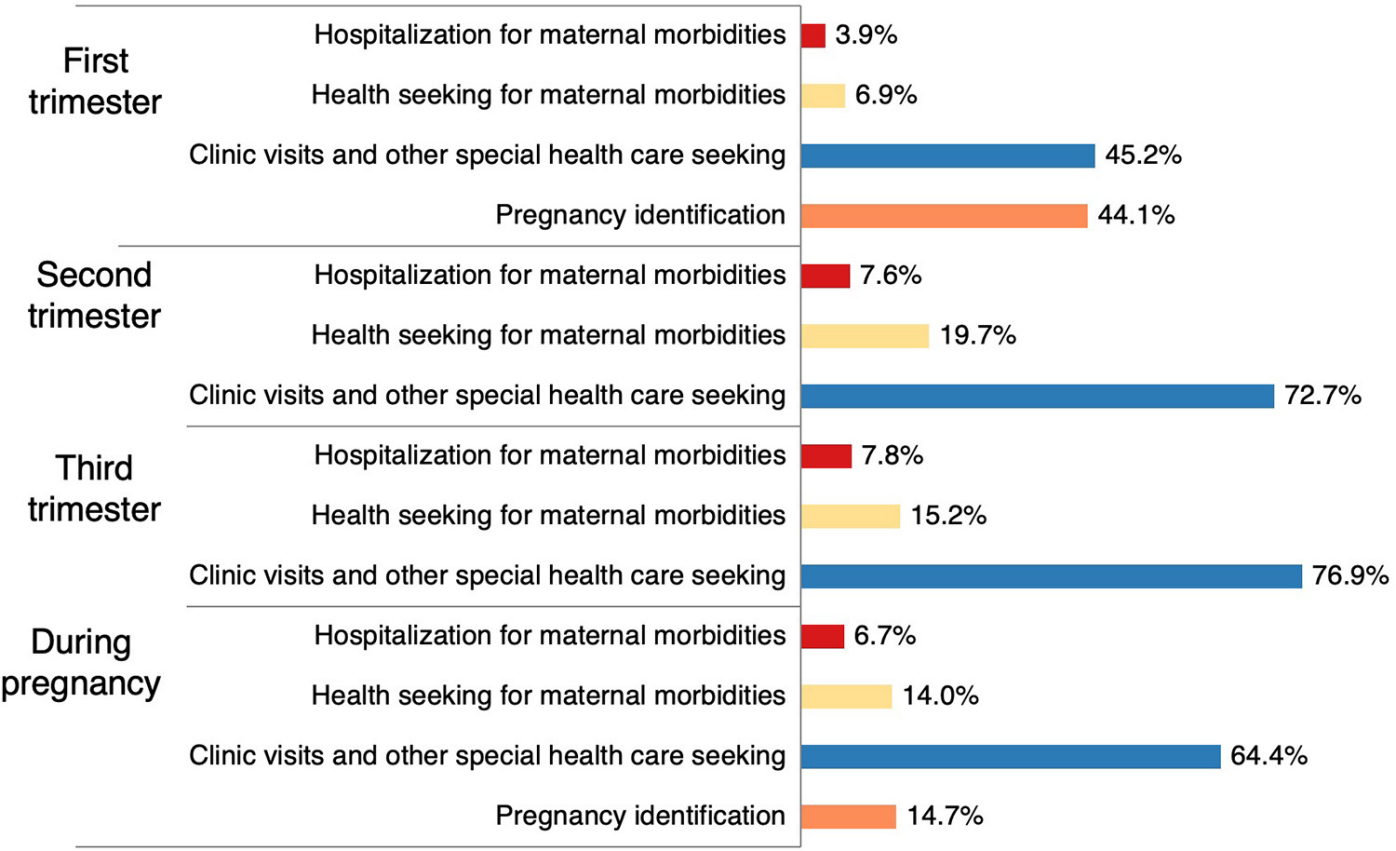
Distribution of Out-of-Pocket Expenditure for Antenatal Care in Health Care Facilities, by Expenditure Events, Anuradhapura District, Sri Lanka

**FIGURE 5:**
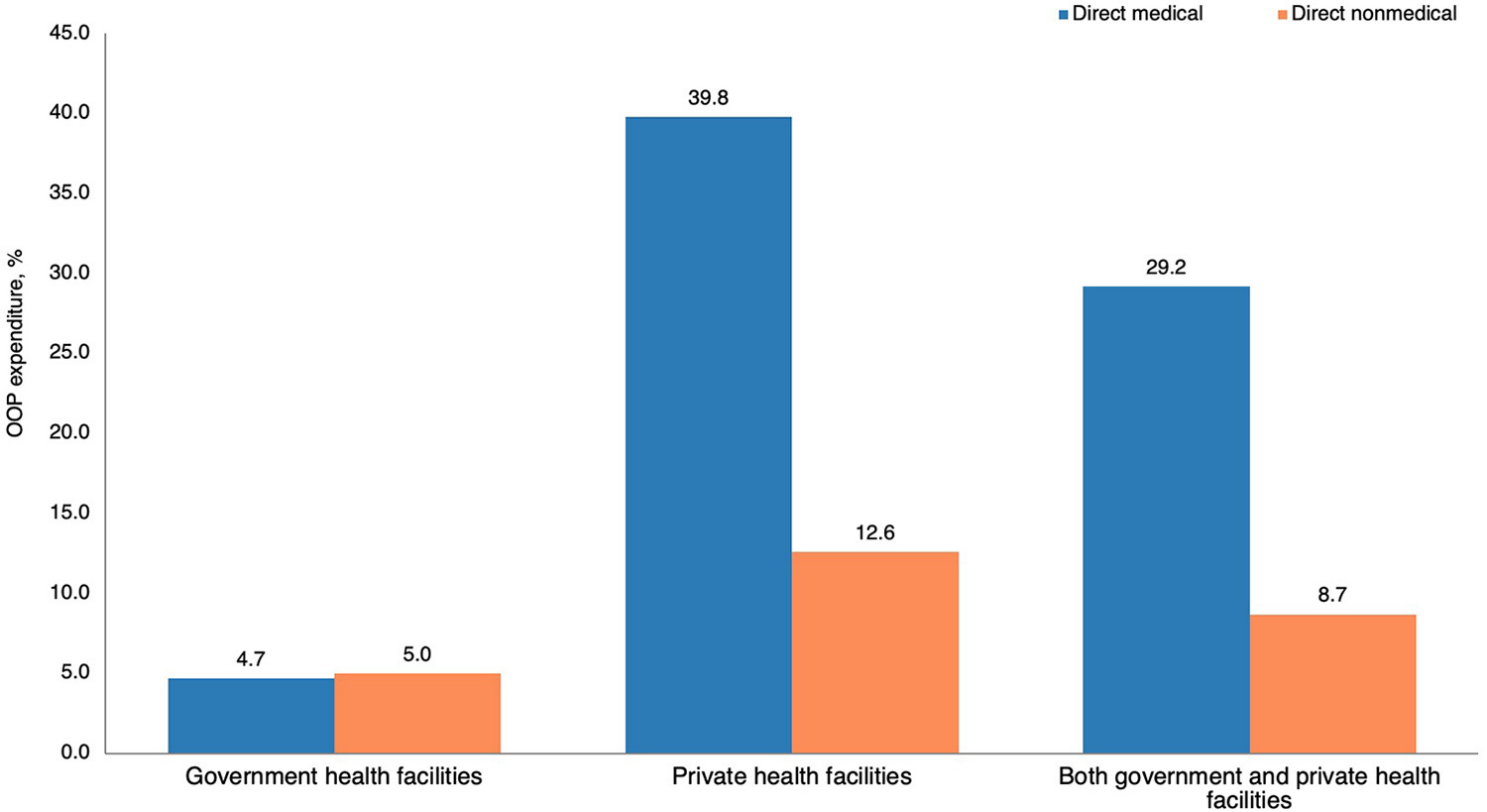
Distribution Within Direct Medical and Nonmedical Out-of-Pocket Expenditure for Antenatal Care in Health Care Facilities, by Health Sector, Anuradhapura District, Sri Lanka

**FIGURE 6:**
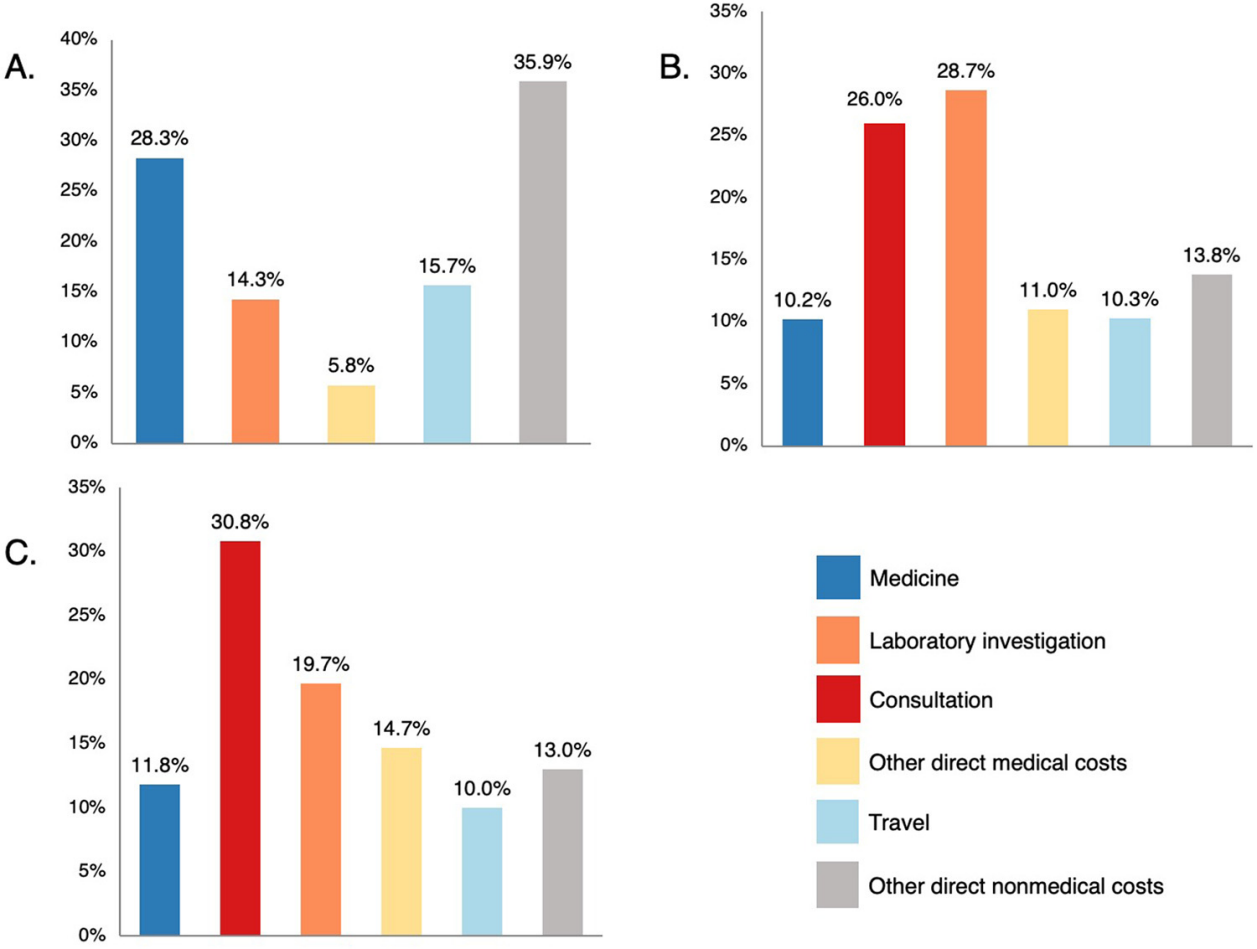
Distribution of Out-of-Pocket Expenditure, by Different Health Care Sector, Anuradhapura District, Sri Lanka: (A) Government Health Care; (B) Private Health Care; (C) Both Government and Private Health Care^a^ ^a^ Other direct medical costs include hospital charges and medical-related items/equipment purchased from outside. Other direct nonmedical costs include cost for food and refreshment while seeking health care, cost for accompanying person(s), and other direct nonmedical costs.

Figure 4 shows the distribution of OOP expenditure across different expenditure events. The highest share of OOP expenditure was spent on clinic visits and other special health care seeking during pregnancy (64.4% of total OOP expenditure). The highest OOP expenditure (52.4% of total OOP expenditure) occurred among pregnant women who used private health facilities (39.8% for direct medical and 12.6% for direct nonmedical OOP expenditure out of total OOP expenditure) followed by pregnant women who used both (government and private health facilities) and government health facilities (Figure 5). Pregnant women who used government health care services spent primarily on direct nonmedical OOP expenditure (51.6% of OOP expenditure spent at government facilities) (Figure 6A). In private health care services, the direct medical OOP expenditure (75.9% of OOP expenditure spent at private facilities) was higher than the direct nonmedical OOP expenditure (24.1%) (Figure 6B). A similar expenditure pattern was observed among pregnant women who used both government and private health care services (Figure 6C). Mean (SD) and median (IQR) per-visit OOP expenditure incurred at different health care modes during pregnancy and across trimesters are shown in Table 3.

During pregnancy, pregnant women had to spend 8.1%, 73.7%, and 50.4% of per-day income at government, private, and both government and private health care facilities. Further, per visit, OOP expenditure was 13.3%, >100%, and 69.9% of per-day household expenditure incurred at government, private, and both (government and private) health care facilities, respectively. Consequently, 213 (13.8%) families had catastrophic spending, where their OOP expenditure was 23.4% of household expenditure.

There is a statistically significant (χ^2^ [2]=624.39; *P*<.001) difference between per-visit OOP expenditure across the 3 health care modes (government, private, and both government and private health care facilities). The results revealed the highest per-visit OOP expenditure reported in private health facilities during pregnancy. A similar pattern was observed in the first (χ^2^ [2]=819.86; *P*<.001), second (χ^2^ [2]=570.37; *P*<.001), and third (χ^2^ [2]=242.75; *P*<.001) trimesters, reporting the highest in private health facilities across all 3 trimesters.

### Magnitude of Total OOP Expenditure During Pregnancy and in Different Trimesters

We used per-visit OOP expenditure and the number of health visits to estimate the total OOP expenditure during pregnancy and in different trimesters. The mean (SD) and median (IQR) total OOP expenditure during pregnancy (using the average exchange rate) were US$57.74 (US$80.96) and US$35.34 (US$14.58–US$71.22), respectively. The mean (SD) and median (IQR) total OOP expenditure during pregnancy, with the highest exchange rate reported in the study period, were US$54.92 (US$77.00) and US$33.62 (US$13.87–US$67.74), respectively. The mean (SD) and median (IQR) total OOP expenditure during pregnancy, with the lowest exchange rate reported in the study period, were US$59.81 (US$83.86) and US$36.61 (US$15.11–US$73.77), respectively.

There was a statistically significant difference in estimated OOP expenditure across 3 trimesters (*P*<.001), and the OOP expenditure estimates were increased across trimesters (Table 4). Furthermore, there was a statistically significant difference in OOP expenditure incurred at private, government, and both health care services (*P*<.001), reporting the highest OOP expenditure in private health care services during pregnancy (Table 4).

**TABLE 4.** Estimated Total Out-of-Pocket Expenditure During Pregnancy and in Different Trimesters Among Rajarata Pregnancy Cohort Study Sample Participants in Anuradhapura District, Sri Lanka

	Number of Visits, Median (IQR)	Estimated OOP Expenditure (US$)		χ^2^ [2]	*P* Value
		Mean (SD)	Median (IQR)		
First trimester	3 (2–3)	18.90 (25.77)	11.70 (2.74–25.91)	20.997^a^	<.001^c^
Second trimester	3 (3–5)	34.39 (50.40)	18.47 (5.90–40.18)		
Third trimester	6 (3–6)	57.68 (96.14)	27.43 (3.02–71.15)		
During pregnancy	11 (9–14)	57.74 (80.96)	35.34 (14.58–71.22)	–	–
First trimester					
Government health facilities	3 (2–3)	4.82 (6.97)	2.16 (0.77–5.66)	699.046^b^	<.001^c^
Private health facilities	3 (3–4)	55.75 (43.28)	46.08 (29.78–63.47)		
Both government and private health facilities	3 (2–3)	24.57 (22.86)	19.79 (11.70–30.45)		
Second trimester					
Government health facilities	3 (3–5)	13.56 (30.26)	4.29 (1.78–11.97)	461.821^b^	<.001^c^
Private health facilities	3 (3–3)	54.23 (56.35)	34.17 (26.33–55.22)		
Both government and private health facilities	4 (3–6)	47.24 (56.19)	27.82 (15.09–57.86)		
Third trimester					
Government health facilities	3 (3–3)	13.88 (42.58)	3.43 (1.17–10.30)	258.906^b^	<.001^c^
Private health facilities	6 (6–6)	83.51 (79.42)	60.34 (46.99–81.73)		
Both government and private health facilities	9 (7–9)	139.82 (148.11)	95.32 (47.43–166.03)		
During pregnancy					
Government health facilities	10 (8–14)	16.08 (28.83)	7.29 (2.64–19.37)	483.306^b^	<.001^c^
Private health facilities	14 (8–30)	115.69 (110.32)	93.33 (46.25–131.48)		
Both government and private health facilities	11 (9–14)	64.92 (77.12)	43.67 (23.51–78.66)		

Abbreviations: IQR, interquartile range; OOP, out-of-pocket; SD, standard deviation; US$, United States dollar.

a Friedman test.

b Kruskal-Wallis test.

c Significant at 0.01 of significance level.

### Associated Factors of Per-Visit OOP Expenditure

Associated factors of per-visit OOP expenditure during pregnancy were initially assessed using bivariate analysis, and 14 factors emerged as significant predictors of OOP expenditure (Supplement Table S2). They were included in the multivariable analysis (Model I) to determine the independent predictors of OOP expenditure. The final model with 9 predictors is shown in Table 5, which was statistically significant (F=33.838; *P*<.001), and the R-square and adjusted R-square values were 0.48 and 0.24, respectively. The Durbin-Watson value of 2.01 confirmed that the model has no autocorrelation issue. The model has no multicollinearity, as the VIF values were lower than 10.

**TABLE 5.** Multivariable Analysis to Determine Independent Predictors of Out-of-Pocket Expenditure for Antenatal Care, Anuradhapura District, Sri Lanka

Independent Predictors	Overall Model		Low-Income Group		Middle-Income Group		High-Income Group	
	Coefficient (95% CI)	T-Statistic (*P* Value)	Coefficient (95% CI)	T-Statistic (*P* Value)	Coefficient (95% CI)	T-Statistic (*P* Value)	Coefficient (95% CI)	T-Statistic (*P* Value)
Constant	−0.95 (−2.27, 0.37)	−1.418 (.157)	0.77 (−1.09, 2.63)	0.819 (.414)	−0.25 (−2.88, 2.38)	−0.187 (.852)	−1.73 (−10.09, 6.63)	−0.407 (.685)
Monthly household income	0.002 (0.001, 0.003)^a^	3.441 (.001)	−0.004 (−0.014, 0.006)	−0.713 (.476)	−0.001 (−0.009, 0.007)	−0.248 (.804)	0.002 (0.001, 0.004)^a^	3.197 (.002)
Monthly household expenditure	0.002 (0.001, 0.004)^b^	1.809 (.071)	0.001 (−0.003, 0.004)	0.272 (.786)	0.001 (−0.003, 0.004)	0.315 (.753)	0.006 (0.002, 0.010)^a^	2.974 (.003)
Number of previous pregnancies	24.315 (−26.704, 75.334)	0.935 (.350)	−25.638 (−108.405, 57.129)	−0.609 (.543)	−68.891 (−142.727, 1.508)^b^	1.838 (.067)	−21.795 (−147.639, 104.049)	−0.341 (.733)
Duration of the marriage	−8.784 (−20.513, 2.945)	−1.469 (.142)	−10.815 (−30.256, 8.626)	−1.095 (.275)	−7.045 (−23.957, 9.868)	−0.818 (.414)	−15.620 (−43.964, 12.724)	−1.085 (.279)
Used health care mode (compared to government health care facilities)								
Private health care facilities	5.95 (4.67, 7.22)^a^	9.144 (.001)	3.78 (1.16, 6.40)^a^	2.840 (.005)	5.84 (4.11, 7.57)^a^	6.625 (<.001)	9.02 (6.01, 12.03)^a^	5.898 (<.001)
Both government and private health care facilities	3.71 (3.20, 4.21)^a^	14.314 (.001)	3.41 (2.62, 4.20)^a^	8.528 (<.001)	3.85 (3.09, 4.62)^a^	9.938 (<.001)	3.99 (2.80, 5.19)^a^	6.570 (<.001)
Having maternal morbidities (compared to no maternal morbidities)								
Having 1 maternal morbidity	0.17 (−0.95, 1.28)	0.292 (.770)	0.58 (−0.65, 1.81)	0.922 (.357)	-	-	−1.50 (−9.65, 6.65)	−0.363 (.717)
Having >1 maternal morbidity	1.65 (0.60, 2.69)^b^	3.085 (.002)	2.03 (1.01, 3.04)^a^	3.931 (<.001)	1.26 (0.45, 2.07)^a^	3.042 (.002)	0.42 (-7.62, 8.45)	0.102 (.919)
Ethnicity (compared with Sinhalese)								
Others (Tamil, Moor, Malay, and others)	−1.12 (−3.06, 0.82)	−1.135 (.257)	−2.37 (−6.06, 1.32)	−1.265 (.207)	−1.01 (−4.11, 2.09)	−0.640 (.523)	0.26 (−3.44, 3.96)	0.140 (.889)
Religion (compared with Buddhist)								
Others (Hindu, Catholic/Christian, Islam)	1.11 (−0.71, 2.94)	1.195 (.232)	2.41 (−1.25, 6.07)	1.294 (.197)	0.77 (−2.09, 3.62)	0.526 (.599)	0.98 (−2.29, 4.24)	0.588 (.557)
Having reproductive health education in school (compared with pregnant women received)								
Pregnant women had not received reproductive health education	0.30 (−0.13, 0.73)	1.363 (.173)	0.44 (−0.27, 1.15)	1.224 (.222)	0.39 (−0.24, 1.01)	1.215 (.225)	−0.04 (−1.03, 0.96)	−0.075 (.941)

Abbreviation: CI, confidence interval.

a Statistically significant at 0.01 of *P* value.

b Statistically significant at 0.1 of *P* value.

Among the 9 predictors, only 4 variables (monthly household income, monthly household expenditure, used health care mode, and having maternal morbidities) had statistically significant associations with OOP expenditure. According to the analysis, OOP expenditure increases by 0.002 times with the monthly household income and expenditure. Compared to the pregnant women who used only government health care facilities, the pregnant women who used private health care facilities and both government and private health care facilities spent an additional US$5.95 and US$3.71, respectively. Moreover, pregnant women with more than 1 maternal morbidity/ill-health condition spent an additional US$1.65 more than those without maternal morbidities/ill-health conditions.

### Associated Factors of Per-Visit OOP Expenditure in Different Income Groups

Bivariate analyses within 3 income groups were conducted separately to assess the significant associated factors of OOP expenditure (Supplement Tables S3, S4, and S5). All of the variables used in Model I were tested to assess the independent predictors of the OOP expenditure (Table 5) in different income groups. All 3 models were statistically significant (*P*<.001) and confirmed that the models have no autocorrelation issue. The R-square values were 0.28, 0.20, and 0.31 in low (Model II), middle (Model III), and high (Model IV) income groups, respectively.

In the model on low-income groups, 3 variables were statistically significant. Compared to the pregnant women who used only government health care facilities, the pregnant women who used private health care facilities and those who used both government and private health care facilities spent an additional US$3.78 and US$3.41, respectively. Further, pregnant women with more than 1 maternal morbidity/ill-health condition spent US$2.03 more than their counterparts.

In the model for the middle-income group, 4 variables were statistically significant. The OOP expenditure decreased by 68.89 times with the number of previous pregnancies. Compared to the pregnant women who used only government health care facilities, the pregnant women who used private health care facilities and those who used both government and private health care facilities spent an additional US$5.84 and US$3.85, respectively. In addition, pregnant women with more than 1 maternal morbidity/ill-health condition spent US$1.26 more than pregnant women without any maternal morbidity.

In the model on the high-income group, 4 variables were statistically significant. The OOP expenditure increased by 0.002 and 0.006 times with the monthly household income and expenditure increase. Compared to the pregnant women who used only government health care facilities, the pregnant women who used private health care facilities and those who used both government and private health care facilities spent an additional US$9.02 and US$3.99, respectively.

### Sources of Financing for Health Care

The pregnant women used different methods for financing OOP expenditure (Table 6). The majority (728 [46.7%]) of pregnant women financed health care using the money they kept for routine transactions during pregnancy, followed by savings (436 [28.0%]), money withdrawn from investment (55 [3.5%]), informal loans (47 [3.0%]), financial assistance (37 [2.4]), selling assets (17 [1.1%]), money from employer (11 [0.7%]), and insurance payments (7 [0.4%]), respectively. The amount spent from any sources was not statistically significant across the 3 trimesters (*P*>.05).

**TABLE 6.** Source of Financing (Per-Visit Basis) for Antenatal Care Among Rajarata Pregnancy Cohort Study Sample Participants, Anuradhapura District, Sri Lanka

	First Trimester			Second Trimester			Third Trimester			During Pregnancy		
	No. (%) (N=1,558)	Mean (SD), US$	Median (IQR), US$	No. (%) (N=1,558)	Mean (SD), US$	Median (IQR), US$	No. (%) (N=1,558)	Mean (SD), US$	Median (IQR), US$	No. (%) (N=1,558)	Mean (SD), US$	Median (IQR), US$
Zero-risk financing^a^												
Financial aid	9 (0.6)	3.67 (1.43)	3.99 (2.99–4.39)	26 (1.7)	5.84 (4.29)	5.49 (3.66–7.31)	4 (0.3)	15.22 (14.24)	16.46 (3.02–27.43)	37 (2.4)	6.53 (6.28)	4.57 (3.66–7.13)
Low-risk financing^b^												
Employer	7 (0.4)	34.91 (37.83)	24.68 (7.48–44.22)	4 (0.3)	54.85 (74.24)	25.60 (10.06–99.65)	1 (0.1)	15.09	15.09	11 (0.7)	41.27 (51.35)	24.68 (7.48–44.22)
Insurance	1 (0.1)	15.09	15.09	5 (0.3)	15.29 (7.62)	14.26 (10.97–16.46)	2 (0.1)	6.40 (1.29)	6.40 (5.49–7.31)	7 (0.4)	16.21 (5.32)	15.09 (13.53–16.46)
Savings	191 (12.3)	10.41 (16.42)	4.99 (2.44–10.97)	248 (15.9)	19.02 (28.18)	9.14 (5.49–21.94)	76 (4.9)	23.18 (29.54)	14.26 (6.17–27.43)	436 (28.0)	15.51 (21.91)	8.91 (4.05–16.53)
Moderate-risk financing^c^												
Money kept for routine transactions	344 (22.1)	9.01 (17.69)	5.3 (2.44–10.70)	446 (28.6)	14.41 (18.85)	9.14 (3.66–16.46)	109 (7.0)	17.98 (20.68)	10.97 (5.49–27.43)	728 (46.7)	12.54 (18.51)	7.31 (3.66–13.71)
High-risk financing^d^												
Money withdrawn from investments	17 (1.1)	22.42 (24.64)	10.97 (5.49–37.61)	34 (2.2)	32.50 (93.16)	9.14 (5.49–19.20)	9 (0.6)	21.45 (28.09)	10.97 (2.74–27.43)	55 (3.5)	29.71 (74.73)	10.97 (6.40–27.43)
By selling assets	7 (0.4)	27.89 (23.84)	19.36 (12.07–44.19)	11 (0.7)	39.06 (27.47)	34.74 (16.91–57.60)	2 (0.1)	69.94 (32.97)	69.94 (46.63–93.25)	17 (1.1)	34.70 (26.37)	27.43 (13.17–54.85)
Informal loans	15 (1.0)	24.72 (49.11)	10.13 (6.40–14.69)	26 (1.7)	47.85 (100.82)	13.71 (3.66–36.57)	10 (0.6)	81.49 (149.93)	19.79 (13.71–54.85)	47 (3.0)	40.24 (81.32)	13.71 (8.69–33.83)

Abbreviations: IQR, interquartile range; SD, standard deviation; US$, United States dollar.

a Non-impact on income, expenditure, and wealth of the household.

b Have an impact on wealth.

c Have an impact on household expenditure pattern.

d Have an impact on income and wealth. (This classification was made purposefully on evidence from the literature).

## DISCUSSION

This study focused on determining the magnitude of OOP expenditure for ANC and the associated factors of OOP expenditure during pregnancy in the Anuradhapura district, Sri Lanka. According to the findings, the average OOP expenditure for ANC during pregnancy and for a single ANC visit was US$57.74 and US$4.18, respectively. We also provide a detailed breakdown of the OOP expenditure across 3 trimesters, different ANC events, and different health care services. Furthermore, we found that even in a predominantly rural district in Sri Lanka, approximately one-fourth of pregnant women used free health care in the government sector only, while those who used private health care services had spent substantial OOP expenditure for ANC. Moreover, the study reveals that monthly household income, expenditure, used health care mode, maternal morbidities, and number of previous pregnancies are independent predictors of OOP expenditure for ANC.

The per-visit OOP expenditure for ANC of the present study (US$4.18) is within the range of the magnitude of OOP expenditure for a single ANC visit (US$2.65–US$194.37) reported in the recent systematic review and meta-analysis conducted in LMICs.^25^ However, it is much lower than the OOP expenditure for a single ANC visit (US$12.93) in other LMICs. This could be due to the country-specific sociopolitical and cultural priority for maternal care. In addition, not all countries in the systematic review provide free maternal health services, which may have led to a higher magnitude due to the paid health service utilization.^25^ Further, compared with the other countries that practice free maternal health care/national programs for reducing maternal OOP expenditure, the present study still shows a lower OOP expenditure than in Ghana,^27^ Malawi, Uganda,^57^ Bangladesh,^58^ and Argentina.^59^ In Ghana, despite having free maternal health services, pregnant women need to make payments due to the unavailability of drugs and other essential supplies in the health facilities, which leads to their outside purchase.^6^^,^^27^^,^^60^ In Uganda,^61^ Bangladesh,^62^ and Argentina,^63^ the higher OOP expenditure is caused by moving toward private health facilities due to weak public health systems, insufficient financial and human resources, poor organization of health services, and lack of information about the local disease burden. However, Sri Lankan maternal health care is considered to be well developed, and pregnant women in Sri Lanka use the government health system more often than the private health system during pregnancy, where the required investigations, medicine, and counseling are provided free of charge.^39^ This could be the reason for the observed lower OOP expenditure compared with other LMICs. As in the per-visit OOP expenditure, the total OOP expenditure during pregnancy in rural Sri Lanka (US$57.74) is also lower than the pooled estimate of US$63.29 reported in LMICs.^25^ Further, we note that the value is even lower than the pooled estimate of US$60.83 in the Southeast Asia Region.^25^ Even though the magnitude of the present study is lower, it is within the range of OOP expenditure for LMICs (US$2.41–US$654.32) and the Southeast Asia Region (US$27.11–US$654.32),^25^ and there was no statistically significant difference between the OOP expenditure magnitude of the present study and the OOP expenditure for ANC in LMICs (*P*>.05) and the Southeast Asia Region (*P*>.05).

The per-visit OOP expenditure for ANC in the present study is lower than the OOP expenditure for a single ANC visit in other LMICs.

The total OOP expenditure for ANC during pregnancy was 3.4% of the annual household expenditure, while the OOP expenditure share was almost one-tenth of the household expenditure in the third trimester. According to the national statistics, the household health expenditure share in rural Sri Lanka is 4.7% of the total annual household expenditure.^64^ Even though the reported magnitude was lower than the Sri Lankan statistics, it is noteworthy that this 3.4% was solely for maternal health care. According to the global evidence, the magnitude of the present study is within the range of global estimates—maternal health care costs range between 1% and 5%; however, they can increase by up to 34% of the total annual household expenditure in Asia and Africa.^65^

Notably, the OOP expenditure per visit is approximately half of the per-day household expenditure in rural Sri Lanka. Further, 213 families had catastrophic expenditures for ANC in rural Sri Lanka, a trend also evident in Myanmar^17^ and India.^18^ In addition, 70.1% of daily income was spent per visit in the daily income group, which was an approximately 20% higher share than the employed group in the government or private sector. Moreover, the magnitude of per-visit OOP expenditure is higher among the low-income group than the middle- and high-income groups. The burden could be substantial because only 7 pregnant women had insurance plans to finance their health care.

Approximately 4% of pregnant women had to sell assets and get informal loans to pay their health care bills and nonmedical OOP expenditures. Consequently, low-income households are pushed into a more financially vulnerable status. This phenomenon is reported by a study conducted in Myanmar,^17^ which describes that 1 in 15 women become impoverished and one-fifth incur catastrophic expenditures due to OOP expenditure for ANC.^17^ Furthermore, the existing 91.6% OOP expenditure of per-day household expenditure pushes households into economic hardship, as approximately half of the pregnant women financed health care by drawing money kept for routine household transactions, and one-quarter of pregnant women used savings, respectively. According to global evidence, the low-income group pays for health care from the savings they keep for their basic essentials.^5^^,^^11^^,^^66^^,^^67^ Thus, the economic burden incurred during pregnancy may be more challenging, even after the pregnancy, for a woman in a low-income group in a country like Sri Lanka due to the ongoing COVID-19 pandemic and the unprecedented financial crisis, which affected the country just after the study period and limited household income generation activities.

OOP expenditure spent per visit does not significantly differ across trimesters; however, the total OOP expenditure significantly differs within trimesters due to the number of visits. In each trimester, the cost incurred at clinics and during other special health care-seeking visits contributed to a higher cost than other ANC services. Considering the total pregnancy, the direct medical OOP expenditure comprises the highest share (73.8%), and the cost of consultation leads to the most extensive percentage (39.3%) among the direct medical OOP expenditure. Moreover, it is noteworthy that pregnant women who used only government health facilities spent 28.3% and 14.3% of OOP expenditure on medicine and laboratory investigation, respectively. These expenditures could be due to various reasons, including the lack or unavailability of certain medicines and investigations in the government health care facilities, unwillingness to spend long waiting times for the services, and opting for better quality services at the private health care facilities.

The presence of OOP expenditure while accessing the government free health care services and national-level free health programs is reported in Sri Lanka,^40^ Bangladesh,^3^^,^^68^ India,^5^^,^^69^ and Nepal.^3^^,^^70^ However, the fact that direct medical OOP expenditure is higher than nonmedical OOP expenditure is challenging the aims of the health care system in Sri Lanka. The direct medical OOP expenditure should be at a minimum level^11^^,^^34^^,^^61^^,^^71^^,^^72^ because the country provides free health care services at the time of service delivery and is considered to have a well-developed maternal care package, which has been instrumental in leading the country to the fourth phase of the obstetric transition.^36^^,^^73^ However, as expected, pregnant women who used only government health care services incurred more direct nonmedical OOP expenditure than direct medical OOP expenditure. Yet, the direct medical OOP expenditure was substantial at approximately half of the OOP expenditure share. In Sri Lanka, all the required investigations, medicines, micronutrient supplements, and consultations are free of charge through routine clinics conducted by the MOH and the grassroots-level services provided by PHMs.^39^ However, the findings suggest that a substantial proportion of pregnant women in rural Sri Lanka still prefer to seek health care through paid health facilities instead of or in addition to using free health care services. Similar findings have been reported in previous local literature.^36^^,^^74^ This is a misuse of Sri Lanka's well-developed maternal care package that is designed for better pregnancy outcomes.^39^ Furthermore, it is a sign of overmedicalization, which is a significant issue in maternal health care service provision in Sri Lanka, a country in the fourth phase of obstetric transition that aims to reduce overmedicalization and duplication of services.^75^

Even though direct medical OOP expenditure can be avoided under a freely available public health care system, the types of direct nonmedical OOP expenditure cannot be avoided. The cost of traveling was the most prominent direct nonmedical cost reported in this study. Traveling expenditure is a significant factor in the accessibility of health services, and the importance of reducing traveling expenditure has been acknowledged in studies in Rwanda^76^ and Sri Lanka.^77^

The findings suggest that the OOP expenditure is higher in households with higher monthly household income and expenditure. This finding aligns with the previous evidence indicating the positive association between income level and the ability to pay for health care reported in the studies carried out in Sri Lanka^40^ and India.^32^^,^^60^^,^^71^^,^^78^ Further, there was a negative relationship between the number of previous pregnancies and the magnitude of the OOP expenditure. This could be due to the experience gained in previous pregnancies that may have led to better financial management, as confirmed in the studies in Sri Lanka^40^ and India.^31^ Also, it could be assumed that lower OOP expenditure occurs for subsequent pregnancies due to less anxiety, concern, and lack of time to pay attention to health seeking.

Moreover, the present study reports that pregnant women with 1 or more maternal morbidities/ill-health conditions tend to have more OOP expenditure compared with pregnant women who do not report any ill-health conditions, which is in accordance with the previous literature.^78^ However, compared with the range of OOP expenditure for maternal morbidities during total pregnancy in LMICs reported in the recent meta-analysis (US$125.98–US$523.03),^25^ the OOP expenditure for maternal morbidities during pregnancy (US$36.68) reported in the present study is substantially low. Further, it is much lower than the OOP expenditure reported in India (US$262.25)^79^ and Ethiopia (US$125.98).^80^ This could be due to the fact that only 427 (29.9%) pregnant women had sought health care despite reporting maternal morbidities/ill health conditions. Among them, the number of pregnant women who used only private health care (73 [5.1%]) was less than those who used government health care (354 [24.8%]) for maternal morbidities. One of the main reasons for this could be that in Sri Lanka, although outpatient care for maternal morbidities would be considered in the private health sector, inpatient care for morbidities would be obtained through the government sector. This tendency is high in the districts such as Anuradhapura, which are predominantly rural.

### Strengths and Limitations

One of the main strengths of the present study is the relatively large sample size in comparison to other global studies that assessed the economic burden of maternal health care. Further, the present study reports a detailed analysis of almost all direct medical and nonmedical OOP expenditure types. In addition, the OOP expenditure throughout the antenatal period is presented in this study, as opposed to the limited periods reported in most of the global literature assessing OOP expenditure for maternal health care. The use of prospective data collection and the adoption of several measures to increase the completeness and accuracy of data were instrumental in improving the data quality.

Even in the context of these strengths, it is essential to note the lower response rate with loss to follow-up, primarily related to the third-trimester data. This could lead to a nonrepresentative sample in all 3 trimesters. Even though many measures were adopted to encourage completion of the self-administered questionnaires immediately after a health care visit, the possibility of recall bias could not be eliminated. Several laboratory and other investigations were provided by the RaPCo study free of charge for pregnant women, which could have led to a potential underestimation of the OOP expenditure in pregnant women who would have used private health care facilities if services were not provided. Furthermore, the total OOP expenditure estimate could be liable to an underestimation or overestimation because it was estimated based on per-visit OOP expenditure and the average number of visits. In addition, the present study did not assess the indirect cost and loss in terms of productivity losses due to absenteeism and presenteeism. Also, the MLRM reported a lower R-squared value, indicating that the predictor variables explain only 48.0% of the variance in the outcome we are trying to predict. Moreover, the findings of this study can be generalized only to the rural population, as the majority of the population of the study setting lives in rural areas.

## CONCLUSION

The study highlights that the OOP expenditure for ANC is high in rural Sri Lanka, and the low-income group reports a higher OOP expenditure for ANC. The direct medical OOP expenditure, which is almost 3 times higher than nonmedical OOP expenditure, is incurred by pregnant women who use only free maternal health care services provided by the government. Three-quarters of pregnant women use both government and private health care facilities. Compared to pregnant women who use government health care services, those who use private and both government and private health care services have significantly higher OOP expenditure. Household income, expenditure, maternal morbidities, used health care mode, and the number of previous pregnancies are significant predictors of OOP expenditure.

In this context, it is of utmost importance to increase the utilization of maternal health care services provided by the government to minimize the additional economic burden of maternal OOP expenditure, especially for low-income groups. Furthermore, to minimize the direct medical OOP expenditure for pregnant women using free government health care services, prompt attention is needed on policy implications to ensure the availability of adequate medicines and essential services, such as basic laboratory investigations in government health care facilities. Future research needs to focus on the impact of high OOP expenditure incurred during pregnancy on the household economies of pregnant women.

## Supplementary Material

GHSP-D-22-00410-supplement.pdf

## Notes

**Peer Reviewed**

**First published online:** October 26, 2023.
