# Supplementary material for: Out-of-Pocket Expenditure for Antenatal Care Amid Free Health Care Provision: Evidence From a Large Pregnancy Cohort in Rural Sri Lanka
Source: Glob Health Sci Pract. 2023 Oct 30;11(5):e2200410. doi: 10.9745/GHSP-D-22-00410 (PMC10615247; doi:10.9745/GHSP-D-22-00410)
Supplement: GHSP-D-22-00410-supplement.pdf [file GHSP-D-22-00410-supplement.pdf]

Supplement to: Gunarathna SP, Wickramasinghe ND, Agampodi TC, Prasanna IR, Agampodi SB. Out-of-pocket expenditure for antenatal care amid free health care provision: evidence from a large pregnancy cohort in rural Sri Lanka. *Glob Health Sci Pract*. 2023;11(5):e2200410. https://doi.org/10.9745/GHSP-D-22-00410

Supplement Table S1. Magnitude of per visit OOPE during pregnancy

Expenditure event	Statistics	Expenditure Breakdown									Cost incurred in different health care mode			Total
		Direct medical OOPE					Direct non-medical OOPE							
		Medicine/ micronutrient supplements	Consultation	Laboratory investigation	Hospital charges	Other direct medical costs*	Traveling	Foods and refreshments	Accompanying persons	Other direct non-medical costs	Government health care	Private health care	Both Government and private health facilities****	
First trimester														
Pregnancy identification	n (%)[1]	-	-	596 (38.3)**	-	861 (55.3)***	549 (35.2)	365 (23.4)	195 (12.5)	40 (2.6)	734 (47.1)	596 (38.3)	-	1,130 (72.5)
	Mean (SD), US$	-	-	16.45 (10.34)	-	0.31 (0.21)	2.73 (4.85)	2.31 (4.63)	1.75 (1.88)	4.43 (6.21)	1.00 (3.55)	16.63 (13.12)	-	8.01 (12.02)
	Median (IQR) , US$	-	-	10.97 (8.23-16.46)	-	0.22 (0.16-0.38)	1.10 (0.55-2.74)	1.37 (0.82-2.74)	1.10 (0.55-2.74)	2.19 (0.82-5.49)	0.27 (0.16-0.55)	13.85 (9.27-19.75)	-	1.92 (0.27-13.33)
Clinic visits and other special health care seeking	n (%)[1]	382 (24.5)	289 (18.5)	-	-	-	694 (44.5)	595 (38.2)	262 (16.8)	188 (12.1)	747 (47.9)	289 (18.5)	-	1,036 (66.5)
	Mean (SD), US$	7.47 (9.89)	15.87 (16.55)	-	-	-	2.12 (3.80)	1.52 (1.84)	1.44 (1.74)	3.80 (6.68)	3.38 (6.31)	29.01 (23.31)	-	10.53 (17.67)
	Median (IQR), US$	2.74 (0.60-10.97)	14.26 (10.97-16.46)	-	-	-	1.10 (0.55-1.65)	1.10 (0.55-1.65)	0.82 (0.55-1.65)	0.82 (0.55-5.47)	1.65 (0.82-3.41)	23.59 (16.81-33.46)	-	3.46 (1.10-15.50)
Health seeking for maternal morbidities	n (%)[1]	202 (13.0)	149 (9.6)	76 (4.9)	-	-	234 (15.0)	-	90 (5.8)	43 (2.3)	182 (11.7)	149 (9.6)	-	331 (21.2)
	Mean (SD), US$	2.19 (3.50)	4.17 (6.55)	2.25 (2.37)	-	-	1.12 (1.76)	-	1.22 (2.05)	1.38 (2.38)	2.40 (3.17)	8.26 (10.04)	-	5.04 (7.70)
	Median (IQR), US$	1.10 (0.55-2.32)	2.53 (1.20-4.88)	1.54 (0.47-3.11)	-	-	0.53 (0.24-1.10)	-	0.57 (0.25-1.22)	0.73 (0.39-1.22)	1.20 (0.49-2.93)	4.86 (2.44-10.85)	-	2.35 (0.99-5.61)
Hospitalization for maternal morbidities	n (%)[1]	51 (3.3)	-	22 (1.4)	11(0.7)	83 (5.3)	72 (4.6)	83 (5.3)	-	29 (1.9)	103 (6.6)	11 (0.7)	-	114 (7.3)
	Mean (SD), US$	2.16 (2.56)	-	2.32 (2.10)	2.45 (2.45)	1.68 (1.28)	2.16 (4.64)	3.14 (6.13)	-	6.48 (13.52)	6.98 (7.59)	19.4 (39.60)	-	8.17 (14.29)
	Median (IQR), US$	1.22 (0.80-2.50)	-	1.74 (0.69-2.74)	1.83 (1.13-2.23)	1.23 (1.01-2.19)	0.96 (0.55-1.91)	1.94 (0.96-3.66)	-	2.11 (1.01-3.92)	4.94 (2.93-8.78)	7.31 (6.31-8.83)	-	5.23 (3.13-8.78)
Second trimester														
Clinic visits and other special	n (%)[1]	634 (40.7)	587 (37.7)	-	-	-	795 (51.0)	753 (48.3)	328 (21.1)	99 (6.4)	484 (31.1)	266 (17.1)	319 (20.5)	1,265 (81.2)
	Mean (SD), US$	5.14 (6.72)	10.32 (5.56)	-	-	-	2.29 (2.99)	1.60 (1.72)	1.43 (1.62)	1.93 (2.76)	3.83 (4.77)	25.49 (11.50)	12.16 (6.93)	10.28 (11.19)

Supplement to: Gunarathna SP, Wickramasinghe ND, Agampodi TC, Prasanna IR, Agampodi SB. Out-of-pocket expenditure for antenatal care amid free health care provision: evidence from a large pregnancy cohort in rural Sri Lanka. *Glob Health Sci Pract*. 2023;11(5):e2200410. https://doi.org/10.9745/GHSP-D-22-00410

Expenditure event	Statistics	Expenditure Breakdown									Cost incurred in different health care mode			Total
		Direct medical OOPE					Direct non-medical OOPE							
		Medicine/ micronutrient supplements	Consultation	Laboratory investigation	Hospital charges	Other direct medical costs*	Traveling	Foods and refreshments	Accompanying persons	Other direct non-medical costs	Government health care	Private health care	Both Government and private health facilities****	
health care seeking	Median (IQR), US$	2.74 (1.06-6.58)	10.06 (5.49-14.26)	-	-	-	1.10 (0.64-2.74)	1.10 (0.55-1.83)	0.91 (0.55-1.83)	0.91 (0.37-2.19)	2.47 (1.10-4.81)	22.76 (17.55-31.63)	10.70 (7.34-14.63)	6.31 (1.65-15.54)
Health seeking for maternal morbidities	n (%)[1]	298 (19.1)	160 (10.3)	109 (7.0)	-	-	362 (23.2)	-	127 (8.2)	47 (3.0)	96 (6.2)	48 (3.1)	12 (0.8)	454 (29.1)
	Mean (SD), US$	3.58 (5.50)	6.80 (6.17)	3.20 (3.92)	-	-	1.96 (2.87)	-	1.78 (2.13)	2.05 (3.07)	7.46 (9.98)	5.62 (7.22)	3.50 (4.05)	7.78 (10.77)
	Median (IQR), US$	1.83 (0.88-4.39)	4.98 (2.24-9.33)	1.65 (0.91-3.84)	-	-	0.91 (0.37-2.19)	-	0.91 (0.55-2.01)	1.08 (0.49-2.19)	0.48 (1.38-9.14)	3.36 (1.10-6.58)	1.90 (0.50-5.85)	4.11 (1.37-9.05)
Hospitalization for maternal morbidities	n (%)[1]	67 (4.3)	-	22 (1.4)	10 (0.6)	104 (6.8)	120 (7.7)	85 (5.5)	-	37 (2.4)	138 (8.9)	8 (0.5)	2 (0.1)	152 (9.8)
	Mean (SD), US$	3.50 (4.24)	-	2.82 (3.26)	4.53 (3.87)	2.43 (3.11)	2.72 (2.86)	3.25 (3.03)	-	4.43 (6.55)	8.49 (10.13)	20.51 (10.05)	8.69 (1.13)	8.95 (10.32)
	Median (IQR), US$	2.47 (0.91-4.48)	-	1.74 (1.24-3.02)	2.74 (1.83-6.86)	1.51 (0.91-2.74)	1.83 (0.75-3.47)	2.74 (0.91-4.57)	-	1.83 (0.91-5.49)	4.59 (2.38-9.87)	19.65 (15.13-25.37)	8.69 (7.89-9.48)	5.03 (2.42-11.10)
Third trimester														
Clinic visits and other special health care seeking	n (%)[1]	185 (11.9)	195 (12.5)	-	-	-	301 (19.3)	251 (16.1)	98 (6.3)	40 (2.6)	224 (14.4)	168 (10.8)	22 (1.4)	414 (26.6)
	Mean (SD), US$	6.49 (8.58)	14.09 (6.25)	-	-	-	2.46 (4.36)	1.78 (1.60)	1.90 (1.93)	2.73 (3.09)	4.56 (7.98)	24.53 (14.17)	13.06 (5.03)	13.12 (14.48)
	Median (IQR), US$	3.29 (1.15-8.23)	13.99 (10.97-16.46)	-	-	-	1.10 (0.55-2.74)	1.37 (0.55-2.26)	1.37 (0.82-2.74)	1.51 (0.55-4.55)	1.92 (0.85-4.53)	20.57 (16.46-29.07)	11.34 (9.08-16.18)	9.60 (1.65-19.47)
Health seeking for maternal morbidities	n (%)[1]	75 (4.8)	36 (2.3)	25 (1.6)	-	-	90 (5.8)	-	28 (1.8)	12 (0.8)	86 (5.5)	33 (2.1)	3 (0.2)	122 (7.8)
	Mean (SD), US$	4.63 (6.09)	7.41 (5.51)	5.01 (3.56)	-	-	2.66 (3.23)	-	2.35 (2.10)	2.74 (2.25)	5.80 (8.14)	16.01 (12.01)	16.70 (8.30)	8.83 (10.39)
	Median (IQR), US$	2.74 (1.17-5.16)	5.35 (3.02-13.71)	4.39 (2.19-6.03)	-	-	1.23 (0.82-2.74)	-	1.94 (1.10-2.74)	2.74 (0.96-3.02)	3.15 (1.37-6.86)	15.91 (5.70-22.22)	21.04 (14.08-21.49)	4.94 (1.65-1.03)
Hospitalization for maternal	n (%)[1]	11 (0.7)	-	2 (0.1)	0	25 (1.6)	27 (1.7)	22 (1.4)	-	9 (0.6)	35 (2.2)	-	-	35 (2.2)
	Mean (SD), US$	3.94 (3.61)	-	1.11 (0.80)	-	6.35 (5.30)	4.75 (6.39)	6.75 (5.37)	-	8.08 (8.89)	15.83 (18.22)	-	-	15.83 (18.22)

Supplement to: Gunarathna SP, Wickramasinghe ND, Agampodi TC, Prasanna IR, Agampodi SB. Out-of-pocket expenditure for antenatal care amid free health care provision: evidence from a large pregnancy cohort in rural Sri Lanka. *Glob Health Sci Pract*. 2023;11(5):e2200410. https://doi.org/10.9745/GHSP-D-22-00410

Expenditure event	Statistics	Expenditure Breakdown									Cost incurred in different health care mode			Total
		Direct medical OOPE					Direct non-medical OOPE							
		Medicine/ micronutrient supplements	Consultation	Laboratory investigation	Hospital charges	Other direct medical costs*	Traveling	Foods and refreshments	Accompanying persons	Other direct non-medical costs	Government health care	Private health care	Both Government and private health facilities****	
morbidities	Median (IQR), US$	2.74 (1.54-5.49)	-	1.11 (0.55-1.67)	-	5.49 (2.74-10.97)	2.74 (1.10-4.39)	5.49 (2.74-8.23)	-	5.49 (2.74-10.97)	9.87 (4.11-17.55)	-	-	9.87 (4.11-17.55)
Per visit OOPE (in direct medical and non-medical OOPE)														
During first trimester	n (%)[1]	1,388 (89.1)					1,141 (73.2)				572 (36.7)	124 (8.0)	672 (43.1)	1,425 (91.5)
	Mean (SD), US$	4.72 (6.69)					1.82 (3.01)				1.59 (2.24)	17.21 (11.01)	8.06 (7.51)	6.05 (7.82)
	Median (IQR), US$	2.93 (0.27-6.92)					0.91 (0.37-1.94)				0.77 (0.27-1.75)	15.23 (11.46-20.18)	6.56 (4.09-9.44)	3.96 (0.91-8.23)
During second trimester	n (%)[1]	866 (55.6)					1,218 (78.2)				453 (29.1)	178 (11.4)	462 (29.7)	1,286 (82.5)
	Mean (SD), US$	6.46 (6.33)					1.80 (2.50)				2.52 (4.14)	13.16 (8.23)	8.96 (7.37)	6.05 (7.32)
	Median (IQR), US$	5.01 (1.89-9.05)					0.94 (0.41-2.15)				1.28 (0.55-2.77)	10.97 (8.78-15.13)	6.74 (4.21-11.39)	3.54 (0.91-8.78)
During the third trimester	n (%)[1]	286 (18.4)					383 (24.6)				221 (14.2)	125 (8.0)	78 (5.0)	424 (27.2)
	Mean (SD), US$	8.01 (6.90)					2.26 (3.58)				2.53 (5.15)	11.47 (6.40)	14.51 (12.37)	7.37 (8.96)
	Median (IQR), US$	7.13 (2.74-10.15)					1.14 (0.46-2.41)				0.96 (0.41-2.47)	9.97 (7.82-13.27)	10.79 (6.63-17.46)	4.98 (0.91-10.50)
During pregnancy	n (%)[1]	1,518 (97.4)					1,494 (95.9)				395 (25.4)	81 (5.2)	1,039 (66.7)	1,558 (100)
	Mean (SD), US$	3.16 (3.39)					1.14 (1.45)				1.07 (1.32)	6.32 (4.387)	5.20 (4.29)	4.18 (4.19)
	Median (IQR), US$	2.38 (0.82-4.33)					0.69 (0.32-1.46)				0.63 (0.26-1.46)	5.06 (3.61-8.11)	4.05 (2.42-6.50)	3.11 (1.35-5.63)

Notes: *The includes any medical-related items; hCG strips for pregnancy identification and syringes/tubes or any other item purchased outside during hospitalization, **The cost here is for pregnancy identification at a health care facility + channeling fees, ***the cost indicated here is the amount spend for hCG strips for self-identification of the pregnancy, ****This denotes the cost incurred for pregnant women who used both government and private health facilities, [1]n(%) denotes the number of pregnant women out of the total sample (1,558 pregnant women)

Supplement to: Gunarathna SP, Wickramasinghe ND, Agampodi TC, Prasanna IR, Agampodi SB. Out-of-pocket expenditure for antenatal care amid free health care provision: evidence from a large pregnancy cohort in rural Sri Lanka. *Glob Health Sci Pract*. 2023;11(5):e2200410. https://doi.org/10.9745/GHSP-D-22-00410

Supplement to: Gunarathna SP, Wickramasinghe ND, Agampodi TC, Prasanna IR, Agampodi SB. Out-of-pocket expenditure for antenatal care amid free health care provision: evidence from a large pregnancy cohort in rural Sri Lanka. *Glob Health Sci Pract*. 2023;11(5):e2200410. https://doi.org/10.9745/GHSP-D-22-00410

Supplement Table S2: Associated factors of OOPE

Socio-demographic, economic, and health Characteristics		n (%)	Per visit OOPE [USD]		Test statistics
Variable	Groups		Mean (SD)	Median (IQR)	
Ethnicity[a,**] (n=1,558, 100%)[1]	Sinhalese	1,339 (85.9)	4.29 (4.33)	3.18 (1.42-5.72)	U = 131,420.00[a] p=0.014
	Other	219 (14.1)	3.48 (3.17)	2.50 (1.01-4.91)	
Religion[a,**] (n=1,558, 100%)[1]	Buddhist	1,325 (85.0)	4.30 (4.34)	3.16 (1.41-5.71)	U = 140,246.50[a] p=0.026
	Other	233 (15.0)	3.52 (3.13)	2.53 (1.09-5.01)	
Education level[b] (n=1,486, 95.4%)[1]	Primary education (up to grade five)	15 (1.0)	4.30 (6.58)	2.11 (0.80-4.14)	χ^2 [3] = 1.818[b] p=0.611
	Junior secondary education (between grade six to nine)	62 (4.2)	4.18 (4.26)	3.09 (0.67-6.29)	
	Senior secondary education (between grade 10 to 13)	1,188 (79.9)	4.18 (4.25)	3.06 (1.41-5.55)	
	Higher education (certificate/diploma/degree)	221 (14.9)	4.35 (4.01)	3.44 (1.58-6.01)	
Status of sexual and reproductive health education[a,*] (n=1,488, 95.5%)[1]	Yes	878 (59.0)	4.48 (4.46)	3.25 (1.50-6.08)	U=244,223.50[a] p=0.004
	No	610 (41.0)	3.81 (3.85)	2.87 (1.25-5.13)	
Engaging income generating activities – pregnant women[a] (n=1,387, 89.0%)[1]	Yes	294 (21.2)	4.55 (4.44)	3.37 (1.57-6.27)	U=154,778.00[a] p=0.334
	No	1093 (78.8)	4.19 (4.09)	3.15 (1.41-5.63)	
Engaging income generating activities – spouse of the pregnant women[a] (n=1,387, 89.0%)[1]	Yes	1,302 (93.9)	4.27 (4.20)	3.18 (1.45-5.71)	U=54,640.50[a] p=0.846
	No	85 (6.1)	4.18 (3.64)	3.31 (1.74-6.27)	
Employment sector – pregnant women[b,***] (n=294, 18.9%)[1]	Government sector	111 (37.7)	5.34 (5.10)	4.46 (1.80-6.96)	χ^2 [2]= 5.256[b] p=0.073
	Private sector	82 (27.9)	4.15 (4.30)	2.98 (1.31-5.06)	
	Other	101 (34.4)	4.01 (3.63)	3.15 (1.56-5.30)	
Employment sector – spouse of the pregnant women[b,**] (n=1,294, 83.1%)[1]	Government sector	421 (32.5)	4.58 (4.18)	3.62 (1.74-6.11)	χ^2 [2]=8.627[b] p=0.013
	Private sector	377 (29.1)	4.20 (4.29)	2.88 (1.47-5.48)	
	Other	496 (38.4)	4.06 (4.15)	3.01 (1.21-5.53)	
Having maternal morbidities/ill health conditions[b,*] (n=1,558, 100%)[1]	No morbidities	101 (6.5)	2.04 (2.48)	1.16 (0.51-3.06)	χ^2 [2]=166.43[b] p<0.001
	Having only one morbidity	286 (18.4)	2.46 (2.90)	1.72 (0.54-3.09)	
	Having more than one morbidity	1,171 (75.2)	4.78 (4.40)	3.76 (1.80-6.25)	
Used health care mode[b,*] (n=1,515, 97.2%)[1]	Government	395 (26.1)	1.07 (1.32)	0.63 (0.26-1.46)	χ^2 [2]=624.39[b] p<0.001
	Private	81 (5.3)	6.32 (4.38)	5.06 (3.61-8.11)	
	Both government and private	1,039 (68.6)	5.20 (4.29)	4.05 (2.42-6.50)	
Income categories[b,*] (n=1,333, 85.6%)[1]	Low (Less than USD164.56)	353 (26.5)	3.76 (3.64)	2.68 (1.19-5.13)	χ^2 [2]=9.165[b] p=0.010
	Middle (From USD 164.57 to USD 318.16)	651 (48.8)	4.32 (4.26)	3.35 (1.56-5.53)	
	High (More than USD 318.17)	329 (24.7)	4.70 (4.56)	3.57 (1.42-6.47)	
Monthly income of the pregnant women[c,**]					r_s=0.132[c] p=0.023

Supplement to: Gunarathna SP, Wickramasinghe ND, Agampodi TC, Prasanna IR, Agampodi SB. Out-of-pocket expenditure for antenatal care amid free health care provision: evidence from a large pregnancy cohort in rural Sri Lanka. *Glob Health Sci Pract*. 2023;11(5):e2200410. https://doi.org/10.9745/GHSP-D-22-00410

Monthly income of the pregnant women's spouse[c,*]	r_s=0.148[c] $p<0.001$
Monthly household income[c,*]	r_s=0.071[c] $p=0.009$
Monthly household expenditure[c,*]	r_s=0.135[c] $p<0.001$
Monthly household savings[c]	r_s=0.044[c] $p=0.424$
Age of the pregnant women[c]	r_s= -0.019[c] $p=0.477$
Duration of the marriage[c,*]	r_s= -0.099[c] $p<0.001$
Number of previous pregnancies[c,*]	r_s= -0.095[c] $p<0.001$

Note: *statistically significant at the p value < 0.01, **statistically significant at the p value < 0.05, ***statistically significant at the p value < 0.1, [a]Mann-Whitney U test, [b]Kruskal-Wallis H test, [c]Spearman's Rank correlation, [1]This denotes the number of pregnant women in terms of the total sample (1,558 pregnant women)

Supplement to: Gunarathna SP, Wickramasinghe ND, Agampodi TC, Prasanna IR, Agampodi SB. Out-of-pocket expenditure for antenatal care amid free health care provision: evidence from a large pregnancy cohort in rural Sri Lanka. *Glob Health Sci Pract*. 2023;11(5):e2200410. https://doi.org/10.9745/GHSP-D-22-00410

Supplement Table S3: Associated factors of OOPE for low income group

Socio-demographic, economic, and health Characteristics		n (%)	Per visit OOPE [USD]		Test statistics
Variable	Groups		Mean (SD)	Median (IQR)	
Ethnicity[a] (n=353, 22.7%)[1]	Sinhalese	284 (80.5)	3.90 (3.73)	2.84 (1.28-5.46)	U = 8,602.50 p=0.116
	Other	69 (19.5)	3.18 (3.21)	2.28 (0.90-4.18)	
Religion[a] (n=353, 22.7%)[1]	Buddhist	283 (80.2)	3.90 (3.73)	2.87 (1.26 -5.46)	U =8,761.50 p=0.135
	Other	70 (19.8)	3.19 (3.18)	2.29 (0.96-4.18)	
Education level[b] (n=338, 21.7%)[1]	Primary education (up to grade five)	3 (0.9)	2.37 (1.54)	2.03 (1.53-3.04)	χ^2 [3] = 5.353 p=0.148
	Junior secondary education (between grade six to nine)	29 (8.6)	2.40 (2.59)	1.64 (0.67-4.18)	
	Senior secondary education (between grade 10 to 13)	275 (81.3)	3.98 (3.78)	2.83 (1.40-5.59)	
	Higher education (certificate/diploma/degree)	31 (9.2)	3.12 (2.87)	2.42 (1.33-3.79)	
Status of sexual and reproductive health education[a,**] (n=339, 21.8%)[1]	Yes	187 (55.2)	4.15 (3.81)	3.10 (1.52-5.99)	U=11,951.50 p=0.012
	No	152 (44.8)	3.28 (3.33)	2.22 (0.96-4.60)	
Engaging income generating activities – pregnant women[a] (n=353, 22.7%)[1]	Yes	26 (7.4)	4.86 (5.30)	3.32 (1.43-5.35)	U= 3,824.50 p=0.394
	No	327 (92.6)	3.67 (3.47)	2.64 (1.19-5.11)	
Engaging income generating activities – spouse of the pregnant women[a] (n=353, 22.7%)[1]	Yes	337 (95.5)	3.71 (3.58)	2.64 (1.19-5.10)	U=2,376.50 p=0.423
	No	16 (4.5)	4.68 (4.73)	3.41 (1.22-5.81)	
Employment sector – pregnant women[b] (n=26, 1.7%)[1]	Government sector	3 (11.6)	8.25 (8.01)	6.27 (3.85-11.66)	χ^2 [2]=2.434 p=0.296
	Private sector	9 (34.6)	4.09 (2.06)	3.85 (3.49-5.10)	
	Other	14 (53.8)	4.62 (6.20)	2.25 (0.89-4.65)	
Employment sector – spouse of the pregnant women[b] (n=335, 21.5%)[1]	Government sector	44 (13.1)	3.86 (3.17)	2.80 (1.60-5.43)	χ^2 [2]=0.993 p=0.609
	Private sector	103 (30.7)	3.78 (3.81)	2.42 (1.38-5.30)	
	Other	188 (56.1)	3.58 (3.50)	2.66 (1.06-4.85)	
Having maternal morbidities/ill health conditions[b,*] (n=353, 22.7%)[1]	No morbidities	61 (17.3)	1.84 (1.85)	1.06 (0.52-2.99)	χ^2 [2]=41.317 p<0.001
	Having only one morbidity	57 (16.1)	2.72 (2.65)	2.05 (0.96-3.57)	
	Having more than one morbidity	235 (66.6)	4.51 (3.95)	3.27 (1.71-6.18)	
Used health care mode[b,*] (n=353, 22.7%)[1]	Government	106 (30.0)	1.14 (1.26)	0.79 (0.29-1.60)	χ^2 [2]=142.488 p<0.001
	Private	8 (2.3)	4.96 (2.77)	3.94 (3.07-7.65)	
	Both government and private	239 (67.7)	4.88 (3.78)	3.86 (2.20-6.25)	
Monthly income of the pregnant women[c,**]					r_s=0.407 p=0.039
Monthly income of the pregnant women's spouse[c]					r_s=0.027 p=0.628

Supplement to: Gunarathna SP, Wickramasinghe ND, Agampodi TC, Prasanna IR, Agampodi SB. Out-of-pocket expenditure for antenatal care amid free health care provision: evidence from a large pregnancy cohort in rural Sri Lanka. *Glob Health Sci Pract*. 2023;11(5):e2200410. https://doi.org/10.9745/GHSP-D-22-00410

Monthly household income[c]	r_s=0.043<br>p=0.425
Monthly household expenditure[c]	r_s=0.073<br>p=0.170
Monthly household savings[c]	r_s=0.102<br>p=0.396
Age of the pregnant women[c]	r_s= -0.084<br>p=0.117
Duration of the marriage[c,***]	r_s= -0.098<br>p=0.077
Number of previous pregnancies[c,*]	r_s= -0.146<br>p=0.006

Note: *statistically significant at the p value < 0.01, **statistically significant at the p value < 0.05, ***statistically significant at the p value < 0.1, [a]Mann-Whitney U test, [b]Kruskal-Wallis H test, [c]Spearman's Rank correlation, [1]This denotes the number of pregnant women in terms of the total sample (1,558 pregnant women)

Supplement to: Gunarathna SP, Wickramasinghe ND, Agampodi TC, Prasanna IR, Agampodi SB. Out-of-pocket expenditure for antenatal care amid free health care provision: evidence from a large pregnancy cohort in rural Sri Lanka. *Glob Health Sci Pract*. 2023;11(5):e2200410. https://doi.org/10.9745/GHSP-D-22-00410

Supplement Table S4: Associated factors of OOPE for middle income group

Socio-demographic, economic, and health Characteristics		n (%)	Per visit OOPE [LKR]		Test statistics
Variable	Groups		Mean (SD)	Median (IQR)	
Ethnicity[a]<br>(n=651, 41.8%)[1]	Sinhalese	586 (90.0)	4.35 (4.34)	3.34 (1.56-5.52)	U = 18,693.00<br>p=0.807
	Other	65 (10.0)	4.05 (3.41)	3.42 (1.61-5.76)	
Religion[a]<br>(n=651, 41.8%)[1]	Buddhist	579 (88.9)	4.38 (4.36)	3.36 (1.57-5.53)	U =19,757.00<br>p=0.470
	Other	72 (11.1)	3.88 (3.34)	3.15 (1.37-5.58)	
Education level[b]<br>(n=620, 39.8%)[1]	Primary education (up to grade five)	7 (1.1)	6.37 (9.09)	3.87 (2.01-5.11)	χ^2 [3] = 2.103<br>p=0.551
	Junior secondary education (between grade six to nine)	13 (2.1)	6.00 (4.56)	6.11 (3.20-9.48)	
	Senior secondary education (between grade 10 to 13)	516 (83.3)	4.29 (4.21)	3.22 (1.57-5.47)	
	Higher education (certificate/diploma/degree)	84 (13.5)	4.43 (4.25)	3.54 (1.30-5.82)	
Status of sexual and reproductive health education[a]<br>(n=621, 39.9%)[1]	Yes	366 (58.9)	4.55 (4.51)	3.36 (1.69-5.76)	U=44,501.00<br>p=0.325
	No	255 (41.1)	4.08 (3.98)	3.38 (1.53-5.28)	
Engaging income generating activities – pregnant women[a]<br>(n=651, 41.8%)[1]	Yes	103 (15.8)	3.86 (3.76)	2.78 (1.53-5.19)	U=25,735.00<br>p=0.156
	No	548 (84.2)	4.41 (4.34)	3.39 (1.57-5.58)	
Engaging income generating activities – spouse of the pregnant women[a]<br>(n=651, 41.8%)[1]	Yes	637 (97.8)	4.34 (4.28)	3.34 (1.56-5.52)	U=4,424.00<br>p=0.960
	No	14 (2.2)	3.81 (2.82)	3.73 (1.88-5.76)	
Employment sector – pregnant women[b]<br>(n=103, 6.6%)[1]	Government sector	15 (14.6)	5.82 (6.05)	3.55 (2.08-7.46)	χ^2 [2]=3.083<br>p=0.214
	Private sector	41 (39.8)	3.29 (3.42)	2.24 (0.55-4.37)	
	Other	47 (45.6)	3.72 (2.90)	3.47 (1.64-5.28)	
Employment sector – spouse of the pregnant women[b]<br>(n=634, 40.7%)[1]	Government sector	251 (39.6)	4.44 (3.98)	3.56 (1.74-5.64)	χ^2 [2]=2.330<br>p=0.312
	Private sector	180 (28.4)	4.37 (4.31)	3.23 (1.51-5.47)	
	Other	203 (32.0)	4.22 (4.65)	3.13 (1.36-5.28)	
Having maternal morbidities/ill health conditions[b,*]<br>(n=651, 41.8%)[1]	No morbidities	-	-	-	χ^2 [1]=65.530<br>p<0.001
	Having only one morbidity	130 (20.0)	2.43 (3.32)	1.70 (0.46-2.93)	
	Having more than one morbidity	521 (80.0)	4.80 (4.34)	3.84 (1.90-6.02)	
Used health care mode[b,*]<br>(n=651, 41.8%)[1]	Government	147 (22.6)	1.06 (1.33)	0.55 (0.26-1.46)	χ^2 [2]=250.179<br>p<0.001
	Private	25 (3.8)	7.06 (3.70)	6.16 (4.28-8.48)	
	Both government and private	479 (73.6)	5.18 (4.36)	4.02 (2.55-6.09)	
Monthly income of the pregnant women[c]					r_s=-0.006<br>p=0.953
Monthly income of the pregnant women's spouse[c]					r_s=0.001<br>p=0.973
Monthly household income[c]					r_s=-0.049

Supplement to: Gunarathna SP, Wickramasinghe ND, Agampodi TC, Prasanna IR, Agampodi SB. Out-of-pocket expenditure for antenatal care amid free health care provision: evidence from a large pregnancy cohort in rural Sri Lanka. *Glob Health Sci Pract*. 2023;11(5):e2200410. https://doi.org/10.9745/GHSP-D-22-00410

	p=0.211
Monthly household expenditure[c,]***	r_s=0.076<br>p=0.053
Monthly household savings[c]	r_s=0.086<br>p=0.274
Age of the pregnant women[c]	r_s=0.005<br>p=0.889
Duration of the marriage[c]	r_s= -0.019<br>p=0.638
Number of previous pregnancies[c]	r_s=0.005<br>p=0.908

Note: *statistically significant at the p value < 0.01, **statistically significant at the p value < 0.05, ***statistically significant at the p value < 0.1, [a]Mann-Whitney U test, [b]Kruskal-Wallis H test, [c]Spearman's Rank correlation, [1]This denotes the number of pregnant women in terms of total sample (1,558 pregnant women)

Supplement to: Gunarathna SP, Wickramasinghe ND, Agampodi TC, Prasanna IR, Agampodi SB. Out-of-pocket expenditure for antenatal care amid free health care provision: evidence from a large pregnancy cohort in rural Sri Lanka. *Glob Health Sci Pract*. 2023;11(5):e2200410. https://doi.org/10.9745/GHSP-D-22-00410

Supplement Table S5: Associated factors of OOPE for high income group

Socio-demographic, economic, and health Characteristics		n (%)	Per visit OOPE [USD]		Test statistics
Variable	Groups		Mean (SD)	Median (IQR)	
Ethnicity[a] (n=329, 21.1%)[1]	Sinhalese	297 (90.3)	4.81 (4.69)	3.57 (1.44-6.58)	U = 4,287.50 p=0.364
	Other	32 (9.7)	3.70 (2.90)	3.28 (1.32-5.48)	
Religion[a] (n=329, 21.1%)[1]	Buddhist	291 (88.4)	4.80 (4.73)	3.55 (1.40-6.68)	U = 5,216.50 p=0.571
	Other	38 (11.6)	3.89 (2.90)	3.73 (1.42-5.60)	
Education level[b] (n=314, 20.2%)[1]	Primary education (up to grade five)	1 (0.3)	-	-	χ^2 [3] = 1.766 p=0.622
	Junior secondary education (between grade six to nine)	9 (2.9)	4.73 (3.68)	5.56 (0.91-8.69)	
	Senior secondary education (between grade 10 to 13)	225 (71.6)	4.66 (4.84)	3.47 (1.26-6.27)	
	Higher education (certificate/diploma/degree)	79 (25.2)	4.94 (4.19)	3.55 (1.76-7.40)	
Status of sexual and reproductive health education[a] (n=312, 20.0%)[1]	Yes	211 (67.6)	5.06 (4.87)	3.72 (1.62-7.20)	U=9,443.00 p=0.104
	No	101 (32.4)	4.14 (4.06)	3.55 (1.20-5.89)	
Engaging income generating activities – pregnant women[a] (n=329, 21.1%)[1]	Yes	165 (50.2)	4.93 (4.66)	3.69 (1.71-6.78)	U=12,614.50 p=0.289
	No	164 (49.8)	4.47 (4.45)	3.49 (1.71-6.78)	
Engaging income generating activities – spouse of the pregnant women[a] (n=329, 21.1%)[1]	Yes	328 (99.7)	4.71 (4.56)	3.63 (1.40-6.47)	U=98.00 p=0.602
	No	1 (0.3)	-	-	
Employment sector – pregnant women[b] (n=165, 10.6%)[1]	Government sector	93 (56.4)	5.17 (4.87)	4.48 (1.79-6.87)	χ^2 [2]=0.862 p=0.650
	Private sector	32 (19.4)	5.25 (5.46)	3.43 (1.68-7.48)	
	Other	40 (24.2)	4.10 (3.28)	3.35 (1.66-5.57)	
Employment sector – spouse of the pregnant women[b] (n=325, 20.9%)[1]	Government sector	126 (38.8)	5.10 (4.80)	4.22 (1.79-7.11)	χ^2 [2]=3.032 p=0.220
	Private sector	94 (28.9)	4.34 (4.75)	3.04 (1.31-5.62)	
	Other	105 (32.3)	4.57 (4.13)	3.37 (1.25-7.04)	
Having maternal morbidities/ill health conditions[b,*] (n=329, 21.1%)[1]	No morbidities	1 (0.3)	-	-	χ^2 [2]=36.456 p<0.001
	Having only one morbidity	56 (17.0)	2.09 (2.35)	1.43 (0.33-2.85)	
	Having more than one morbidity	272 (82.7)	5.24 (4.72)	4.27 (1.86-7.19)	

Supplement to: Gunarathna SP, Wickramasinghe ND, Agampodi TC, Prasanna IR, Agampodi SB. Out-of-pocket expenditure for antenatal care amid free health care provision: evidence from a large pregnancy cohort in rural Sri Lanka. *Glob Health Sci Pract*. 2023;11(5):e2200410. https://doi.org/10.9745/GHSP-D-22-00410

Used health care mode[b,*] (n=329, 21.1%)[1]	Government	65 (19.8)	0.76 (0.84)	0.44 (0.25-1.02)	χ^2 [2]=130.410 p<0.001
	Private	8 (2.4)	10.72 (7.52)	8.15 (6.85-11.13)	
	Both government and private	256 (77.8)	5.51 (4.39)	4.47 (2.59-7.32)	
Monthly income of the pregnant women[c]					r_s=0.093 p=0.235
Monthly income of the pregnant women's spouse[c]					r_s= -0.023 p=0.678
Monthly household income[c,***]					r_s=0.096 p=0.081
Monthly household expenditure[c,*]					r_s=0.223 p<0.001
Monthly household savings[c]					r_s= -0.064 p=0.554
Age of the pregnant women[c]					r_s=0.016 p=0.775
Duration of the marriage[c,*]					r_s= -0.176 p=0.002
Number of previous pregnancies[c,*]					r_s= -0.175 p=0.004

Note: *statistically significant at the p value < 0.01, **statistically significant at the p value < 0.05, ***statistically significant at the p value < 0.1, [a]Mann-Whitney U test, [b]Kruskal-Wallis H test, [c]Spearman's Rank correlation, [1]This denotes the number of pregnant women in terms of total sample (1,558 pregnant women)
